# The Legionella pneumophila IcmSW Complex Interacts with Multiple Dot/Icm Effectors to Facilitate Type IV Translocation

**DOI:** 10.1371/journal.ppat.0030188

**Published:** 2007-12-07

**Authors:** Eric D Cambronne, Craig R Roy

**Affiliations:** Section of Microbial Pathogenesis, Yale University School of Medicine, Boyer Center for Molecular Medicine, New Haven, Connecticut, United States of America; Tufts University School of Medicine, United States of America

## Abstract

Many Gram-negative pathogens use a type IV secretion system (T4SS) to deliver effector proteins into eukaryotic host cells. The fidelity of protein translocation depends on the efficient recognition of effector proteins by the T4SS. Legionella pneumophila delivers a large number of effector proteins into eukaryotic cells using the Dot/Icm T4SS. How the Dot/Icm system is able to recognize and control the delivery of effectors is poorly understood. Recent studies suggest that the IcmS and IcmW proteins interact to form a stable complex that facilitates translocation of effector proteins by the Dot/Icm system by an unknown mechanism. Here we demonstrate that the IcmSW complex is necessary for the productive translocation of multiple Dot/Icm effector proteins. Effector proteins that were able to bind IcmSW in vitro required *icmS* and *icmW* for efficient translocation into eukaryotic cells during L. pneumophila infection. We identified regions in the effector protein SidG involved in *icmSW*-dependent translocation. Although the full-length SidG protein was translocated by an *icmSW*-dependent mechanism, deletion of amino terminal regions in the SidG protein resulted in *icmSW*-independent translocation, indicating that the IcmSW complex is not contributing directly to recognition of effector proteins by the Dot/Icm system. Biochemical and genetic studies showed that the IcmSW complex interacts with a central region of the SidG protein. The IcmSW interaction resulted in a conformational change in the SidG protein as determined by differences in protease sensitivity in vitro. These data suggest that IcmSW binding to effectors could enhance effector protein delivery by mediating a conformational change that facilitates T4SS recognition of a translocation domain located in the carboxyl region of the effector protein.

## Author Summary

Intracellular pathogens often manipulate the activities of the eukaryotic host cell in which they reside by using a specialized transport apparatus known as a type IV secretion system to deliver proteins that directly manipulate host cell processes. How proteins to be delivered into eukaryotic cells are recognized by a type IV section system is not well understood. For Legionella pneumophila, the bacterium that causes a severe pneumonia known as Legionnaires disease, a type IV system called Dot/Icm is used to deliver an estimated 150 different proteins into host cells during infection. In this study, we demonstrate that a complex consisting of the proteins IcmS and IcmW bind many of the substrate proteins transported into eukaryotic host cells by the Dot/Icm system. Binding of the IcmSW complex to Dot/Icm substrate proteins enhanced the efficiency by which the substrate proteins were transported into cells by a process that involved altering the conformation of the substrate protein. Thus, this work defines a step that is important for the type IV secretion process and provides new molecular details on substrate protein recognition by type IV secretion systems.

## Introduction

Many Gram-negative bacteria utilize a type IV secretion system (T4SS) to deliver proteins and DNA into a recipient cell [1]. The Legionella pneumophila Dot/Icm T4SS is an essential virulence determinant that translocates effector proteins into eukaryotic cells [2–4]. Effector proteins translocated by the Dot/Icm system function to delay endocytic maturation of the vacuole in which the bacterium resides, as well as promote remodeling of the L. pneumophila–containing vacuole into an organelle that resembles the endoplasmic reticulum (ER) [5–10]. L. pneumophila replication occurs in the ER-derived vacuole created by these Dot/Icm-dependent processes [2,3,11]. Currently, over 50 effector proteins have been shown to be translocated into eukaryotic cells by the Dot/Icm T4SS, and recent predictions suggest that there could be as many as 150 effectors [12–26].

Several studies have demonstrated that C-terminal regions of effector proteins are required for translocation into recipient cells by the T4SS [13,27–34]. Analysis of Dot/Icm effectors has not revealed significant primary sequence homology in the C-terminal regions of effector proteins. However, C-terminal translocation domains are thought to have shared biophysical properties critical for translocation by the T4SS. Currently, the only crystal structure of a type IV effector available is for the RalF protein of L. pneumophila [35]. RalF has a C-terminal domain of 20 amino acids (aa) that is necessary and sufficient for Dot/Icm-mediated protein translocation into host cells [14,32]. The RalF crystal structure revealed that this C-terminal domain is largely disordered and separated from the protein's globular domains by a long alpha helix [35]. Thus, for RalF there is evidence that a disordered C-terminal translocation domain is available for interaction with T4SS components and that the availability of this C-terminal domain is sufficient for substrate recognition by the Dot/Icm system.

In contrast to RalF, recent studies have identified effectors that require the IcmS and IcmW proteins for efficient translocation [21,25,36]. IcmS and IcmW are small (12 and 18 kDa, respectively) acidic (pI ∼4) cytoplasmic proteins that have physical properties consistent with chaperone molecules found in many type III secretion systems [37]. L. pneumophila Δ*icmS* or Δ*icmW* strains retain the ability to replicate in macrophage-like cell lines, induce contact-dependent formation of pores in macrophage membranes, and secrete the DotA protein into culture supernatant [11,36,38]. All of these activities are abolished in most other *dot* and *icm* mutants, suggesting that IcmS and IcmW are not essential components of the Dot/Icm secretion machinery.

In previous studies, the IcmS and IcmW proteins were found to interact and form a complex in the L. pneumophila cytoplasm [21,39,40]. Additionally, the IcmS protein has been shown to stabilize IcmW in the bacterial cell, indicating that the IcmS–IcmW interaction is biologically significant. There is both genetic and biochemical evidence that IcmS and IcmW associate with a subset of Dot/Icm effectors [21,25], and that translocation of effectors that associate with the IcmSW complex is reduced in Δ*icmS* and Δ*icmW* strains [21]. Interestingly, RalF is currently the only effector translocated into host cells independent of the IcmS and IcmW proteins, and it has been shown that IcmSW does not bind to RalF in vitro [21]. These data indicate that IcmS and IcmW play an important role in the translocation of some, but not all of the effectors of the Dot/Icm T4SS, suggesting that the IcmSW complex might be critical for the type IV recognition of a particular class of effector proteins. Here, we set out to further explore this possibility by investigating how the IcmSW complex contributes to the translocation of type IV substrates.

## Results

### The IcmSW Complex Is Necessary for the Translocation of Multiple Effector Proteins

**Figure 1:**
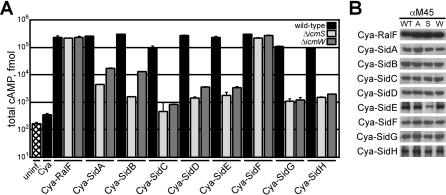
The IcmSW Complex Is Necessary for the Translocation of Multiple Effector Proteins (A) CHO FcγRII cells were left uninfected (hatch), or infected with wild-type (black), Δ*icmS* (light grey), or Δ*icmW* (dark grey) L. pneumophila strains expressing the indicated Cya hybrid proteins. After 1 h of infection, tissue culture cells were lysed and cAMP was extracted from the sample. Total cAMP production induced by translocation of the hybrid was quantified using an enzyme-immunoassay system, indicated as fmol. Results represent average values ± SD of experiments performed in triplicate. (B) Immunoblots of whole-cell bacterial extracts expressing indicated Cya hybrid proteins from wild-type (WT), Δ*dotA* (A), Δ*icmS* (S), and Δ*icmW* (W) probed with monoclonal antibody specific to the M45 epitope.

Previous studies indicated that Dot/Icm effector proteins can be separated into different classes based on their requirement for *icmS* and *icmW*, suggesting that the IcmSW complex may target a particular set of substrates [21]. The *sid* (substrate of *icm-dot* transporter) genes encode a diverse group of proteins transported by the Dot/Icm system, some of which were shown to be translocated by a mechanism involving the IcmSW complex [13,21,25]. These effectors do not demonstrate primary sequence homology. Several of the Sid proteins have regions containing predicted coiled-coil regions and hydrophobic segments that are predicted to represent membrane-spanning regions (Figure S1). To define the repertoire of Sid proteins utilizing IcmSW for translocation by the Dot/Icm system, reporter proteins were constructed that consist of the catalytic domain of the calmodulin-dependent adenylate cyclase (Cya) from Bordetella pertussis fused to the amino terminus of each Sid protein. The production of cyclic adenosine-monophosphate (cAMP) resulting from the translocation of a Cya-Sid hybrid into mammalian cells was used to measure productive translocation of each effector [32,41]. These data show that all of the Cya-Sid fusion proteins were translocated by L. pneumophila into host cells by a Dot/Icm-dependent mechanism (Figure S1). When the Cya-Sid proteins were expressed in either Δ*icmS* or Δ*icmW* strains, a significant decrease in translocation was observed for all hybrids with the exception of Cya-SidF, which was translocated at nearly wild-type levels by either the Δ*icmS* or Δ*icmW* strain (Figure 1A). The *icmS* or *icmW* independence observed for SidF translocation was similar to that shown previously for RalF [21]. The defect in translocation of Sid proteins by *dot* and *icm* mutants was not due to a loss of stability, as steady-state protein levels of the hybrids were similar in wild-type, Δ*dotA*, Δ*icmS*, or Δ*icmW* strains (Figure 1B).

**Figure 2:**
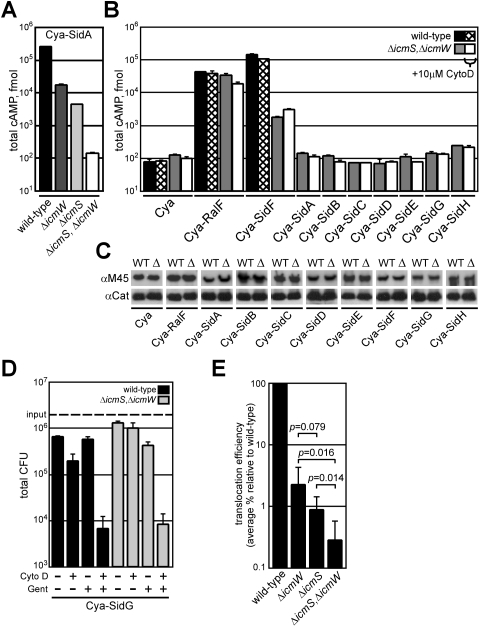
Translocation Defects Observed for the L. pneumophila Δ*icmS*, Δ*icmW* Double Mutant (A) CHO FcγRII cells were infected with wild-type (black), Δ*icmW* (dark grey), Δ*icmS* (light grey), or Δ*icmS*, Δ*icmW* (white) strains expressing Cya-SidA. Total cAMP production was determined as in Figure 1. (B) CHO FcγRII cells were infected with wild-type (black) or Δ*icmS*, Δ*icmW* (grey) strains expressing the indicated Cya hybrid proteins for 1 h. Wild-type (hatch) or Δ*icmS*, Δ*icmW* (white) indicate samples that were conditioned with cytochalasin D prior to infection. Total cAMP production induced by translocation of the hybrid was determined as in (Figure 1). (C) Immunoblots of whole-cell bacterial extracts expressing indicated Cya hybrid proteins from wild-type (WT) or Δi*cmS*, Δ*icmW* (Δ) probed with monoclonal antibody specific to the M45 epitope. Immunoblots for chloramphenicol acetyltransferase (Cat) are included as a load control. (D) CHO FcγRII cells were infected with wild-type (black) or Δ*icmS*, Δ*icmW* (grey) L. pneumophila strains expressing Cya-SidG. Bacteria (3 × 10^6^; MOI = 30) were added to each well (input line) and subjected to the conditions indicated. +/− Cyto D indicates the addition of cytochalasin D (10 μM) 30 min prior to and during infection. +/− Gent indicates the addition of gentamycin (10 μM) 15 min post-infection. After 1 h of infection, tissue culture cells were washed and osmotically lysed. Lysates were serially diluted, plated, and incubated to determine total colony-forming units (CFU) from each condition. Bars represent average values ± SD of experiments performed in triplicate. (E) Graphical representation of the average translocation efficiency of the *icmSW*-dependent substrates SidA, SidB, SidC, SidD, SidE, SidG, and SidH. Translocation of each construct in wild-type is considered 100%. The average % defect is plotted for the Δ*icmS*, Δ*icmW*, and Δ*icmS*, Δ*icmW* strains ± SD of translocation assays performed in triplicate. *p*-Values from Student's *t*-test are indicated as comparisons between strains.

Because the IcmS and IcmW proteins might retain partial activity when they are not in a complex with each other, translocation of the Sid proteins was analyzed in an L. pneumophila strain lacking both *icmS* and *icmW.* The translocation of most Cya fusion proteins was reduced in the Δ*icmS*, Δ*icmW* double mutant compared to either single mutant. An additive translocation defect was observed for Cya-SidA when data for wild-type L. pneumophila was compared to the single mutants Δ*icmS* or Δ*icmW*, and the Δ*icmS*, Δ*icmW* double mutant (Figure 2A). Translocation of SidA, SidB, SidC, SidD, SidE, SidG, and SidH decreased roughly 10-fold in the Δ*icmS*, Δ*icmW* double mutant compared with the single Δ*icmS* mutant (Figure 2B). Although SidF translocation was largely independent of *icmS* or *icmW* in the single mutants, translocation of SidF was reduced by nearly 100-fold in the Δ*icmS*, Δ*icmW* double mutant (Figure 2B). Additionally, IcmSW was isolated in a complex with SidF when the three proteins were co-expressed in *Escherichia coli* (unpublished data). Translocation of RalF was not severely attenuated in the Δ*icmS*, Δ*icmW* double mutant, providing further evidence that RalF is an IcmSW-independent effector (Figure 2B). Equal translocation of SidF by wild-type L. pneumophila in cytochalasin D–treated cells indicates that that delivery of this effector by the Dot/Icm system does not require bacterial internalization (Figure 2B). There was a similar defect in Sid protein translocation by the Δ*icmS*, Δ*icmW* double mutant observed when bacterial uptake was blocked using cytochalasin D (Figure 2B). This result indicated that the defect in effector translocation was not due to differences in the vacuoles that the Δ*icmS*, Δ*icmW* double mutant occupied compared with wild-type L. pneumophila. Fusion protein levels were similar in wild-type L. pneumophila and the Δ*icmS*, Δ*icmW* double mutant, indicating that the translocation defect observed for the double mutant is not due to a difference in steady-state protein levels (Figure 2C). No defect in internalization of the Δ*icmS*, Δ*icmW* double mutant was observed when compared to wild-type L. pneumophila, indicating that translocation differences were not due to differences in uptake of the two strains (Figure 2D).

The translocation efficiency of each Cya-Sid fusion protein by the single Δ*icmS* or Δ*icmW* mutants or the Δ*icmS*, Δ*icmW* double mutant was calculated as the percent of cAMP relative to the wild-type control strain producing the same fusion protein. The average translocation defect for each strain was calculated using these efficiency values (Figure 2E). These data show a significant decrease in Sid translocation in both the Δ*icmW* and Δ*icmS* single mutants. Although the defect observed for the *icmS* mutant was larger than the defect for the Δ*icmW* mutant, this difference was not statistically significant. A significant reduction in translocation efficiency was observed in the Δ*icmS*, Δ*icmW* double mutant when compared with the single mutants. These data demonstrate that a functional IcmSW complex is critical for the efficient translocation of multiple effector proteins and suggests that IcmS and IcmW may retain partial activity when they are not in a complex with the other protein.

### SidG Contains a C-Terminal Translocation Signal

**Figure 3:**
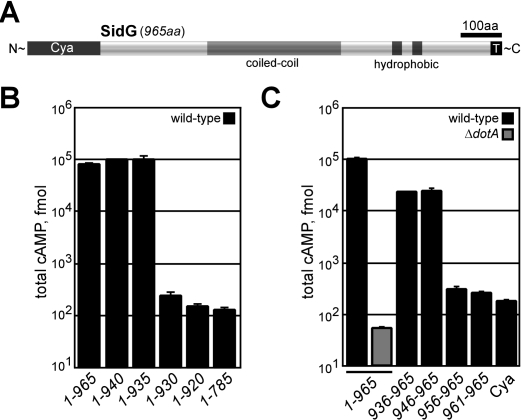
SidG Contains a C-Terminal Translocation Signal (A) Schematic representation of the 965-aa SidG protein, which contains an extensive coiled-coil region (grey) as well as two hydrophobic segments (black). The identified translocation signal is depicted (T). (B and C) CHO FcγRII cells were infected with wild-type (black) or Δ*dotA* (dark grey) strains expressing the indicated Cya-SidG hybrid proteins for 1 h. Total cAMP production induced by translocation of the hybrid was determined as in Figure 1.

The SidG protein demonstrated a strict requirement for *icmS* and/or *icmW* for efficient translocation. SidG contains a predicted coiled-coil region, as well as two distinct hydrophobic segments that may represent membrane-spanning regions (Figure 3A). Deletion analysis was used to map the translocation signal in SidG. Deletion of 35 aa from the SidG C-terminus abrogated translocation of the protein into host cells, demonstrating that this C-terminal region is necessary for Dot/Icm-dependent translocation (Figure 3B). Consistent with this region encoding a translocation signal, a Cya hybrid containing only the C-terminal 20 aa of SidG*_(946–965)_* was sufficient to promote Dot/Icm-mediated protein translocation (Figure 3C). Overall, these data define a C-terminal region in SidG that is necessary and sufficient for Dot/Icm-mediated translocation.

### A Central Region in SidG Confers the *icmSW*-Dependent Translocation Phenotype

**Figure 4:**
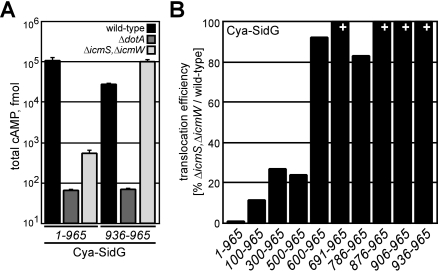
The C-Terminal Translocation Signal of SidG Supports *icmSW*-Independent Translocation (A) CHO FcγRII cells were infected with wild-type (black), Δ*dotA* (dark grey), or Δ*icmS*, Δ*icmW* (light grey) strains expressing indicated Cya-SidG hybrid proteins for 1 h. Total cAMP production induced by translocation of the hybrid was determined as in Figure 1. (B) CHO FcγRII cells were infected with wild-type or Δ*icmS*, Δ*icmW* strains expressing the indicated Cya-SidG hybrid proteins for 1 h. Total cAMP production induced by translocation of the hybrid was determined for each strain. % values represent translocation efficiency as a measure of (Δ*icmS*, Δ*icmW*) / wild-type for the indicated Cya-SidG hybrid proteins. + indicates that cAMP production induced by translocation of the hybrid in Δ*icmS*, Δ*icmW* exceeded levels produced in wild-type.

To investigate whether the IcmSW complex is important for recognition of the C-terminal translocation domain of an *icmSW*-dependent effector, we measured translocation of a Cya hybrid containing a C-terminal 30-aa region of SidG containing the translocation signal. These data show that efficient translocation of the Cya-SidG*_(936–965)_* protein did not require the IcmS and IcmW proteins (Figure 4A). Thus, the IcmSW complex does not contribute directly to the recognition of the C-terminal translocation signal in SidG. These experiments suggest sequences that are N-terminal to the SidG translocation signal confer the *icmSW*-dependent translocation phenotype observed for the full-length fusion protein. To identify sequences necessary for the *icmSW*-dependent phenotype, N-terminal sequences were deleted and translocation efficiencies of the deletion derivatives were compared between wild-type *L. pneumophila* and the Δ*icmS*, Δ*icmW* double mutant. Efficient translocation of Cya-SidG*_(500–965)_* required the IcmSW complex, whereas an *icmSW*-independent translocation phenotype was observed for the Cya-SidG*_(600–965)_* protein (Figures 4B and S2). These data define a central region in the SidG protein as being important for the *icmSW*-dependent translocation phenotype, and indicate that this internal region negatively impacts SidG translocation in the absence of the IcmSW complex.

### IcmS and IcmW Interactions with SidG Are Outside of the Translocation Signal

**Figure 5:**
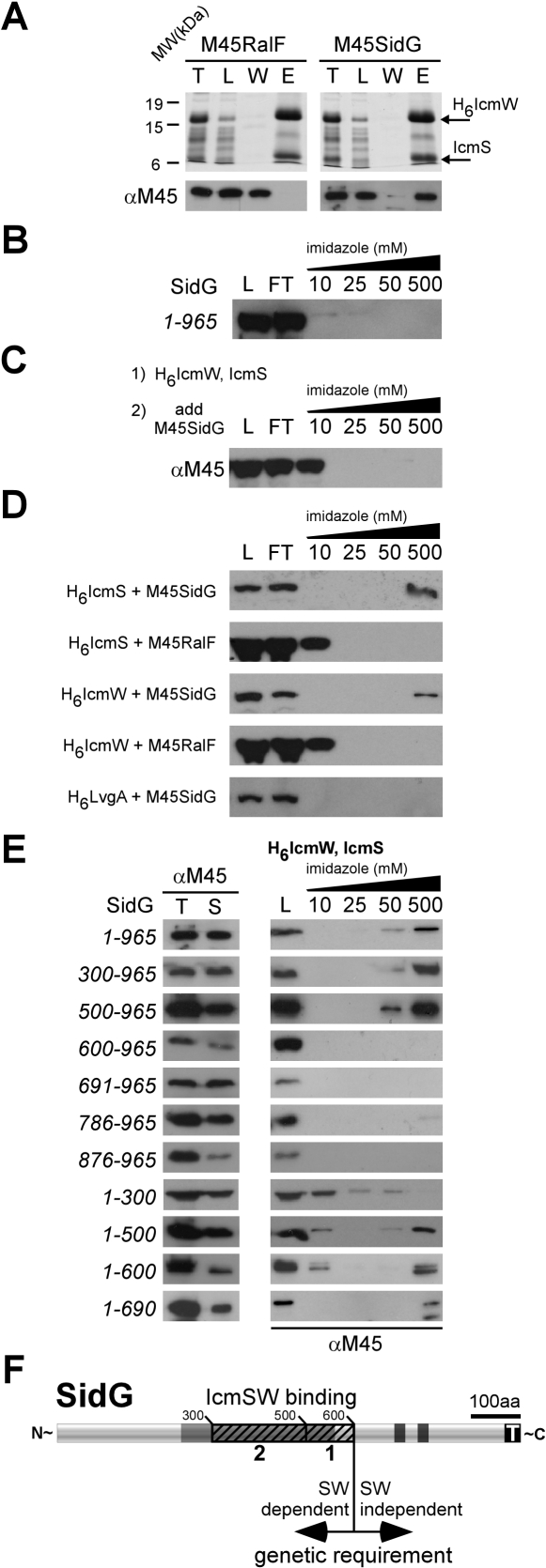
IcmSW Complex Binds to a Region of SidG That Is Distinct from the C-Terminal Translocation Domain (A) E. coli expressing H_6_IcmW/IcmS, and either M45-RalF or M45-SidG were lysed (T), clarified, and loaded (L) onto affinity columns, washed (W), and eluted (E). Fractions were resolved on SDS-PAGE, where H_6_IcmW/IcmS were detected with Coomassie staining, and RalF or SidG was detected by immunoblotting with M45-specific antisera. MW indicators for the gel are shown on the left (kDa). (B) E. coli expressing M45-SidG alone was subjected to affinity purification as in (A). M45-SidG was detected as in (A). (C) Lysate from E. coli expressing H_6_IcmW/IcmS was pre-charged onto affinity column (1), washed, and subjected to lysate from E. coli expressing M45-SidG (2) (L). Flow-through (FT) was collected and the column was washed and eluted with buffer containing the indicated concentration of imidazole. M45-SidG was detected as in (A). (D) E. coli strains expressing H_6_IcmS, H_6_IcmW, or H_6_LvgA and M45-SidG or M45-RalF were subjected to affinity purification as in (B). M45-SidG or M45-RalF was detected as in (A). (E) E. coli expressing H_6_IcmW/IcmS, and indicated M45-tagged SidG truncations were lysed (T), clarified (S), and loaded (L) onto affinity columns, and washed and eluted with buffer containing imidazole in the indicated concentrations. Purified M45-SidG fragments were resolved on SDS-PAGE and detected as in (A). (F) Schematic representation of the genetic (arrows) and biochemical (hatch) contribution of IcmSW on SidG translocation. At least one IcmSW binding site resides between residues 500 and 600 of SidG [1], with additional binding sites located between residues 300 and 500 [2]. The IcmSW binding region is distinct from the C-terminal translocation domain (T).

IcmSW binding to SidG was analyzed by co-purification of recombinant proteins produced in E. coli [21]. N-terminal hexa-histidine-tagged IcmW (H_6_IcmW) and IcmS were produced from a bi-cistronic message in conjunction with M45-epitope-tagged SidG produced from a second vector in the same E. coli cell [21]. H_6_IcmW and associated proteins were captured from lysates on affinity columns. These data show that SidG co-purified with the H_6_IcmW/IcmS complex isolated from E. coli cells producing the three proteins (Figure 5A). RalF did not co-elute with the H_6_IcmW/IcmS complex when produced in the same E. coli cell, as shown previously (Figure 5A) [21]. Additionally, SidG produced in the absence of the IcmSW complex did not bind to the affinity column (Figure 5B). These data demonstrate that SidG contains binding sites that mediate specific interactions with the IcmSW complex.

Further analysis of SidG interactions with the IcmSW complex revealed that H_6_IcmW/IcmS complex immobilized on a column was unable to bind SidG protein contained in E. coli lysates (Figure 5C), suggesting that interactions between SidG and IcmSW occur in the cell before SidG folding has been completed. When M45-SidG was co-expressed in E. coli with either H_6_IcmS or H_6_IcmW, binding of SidG to these individual components of the IcmSW complex was detected (Figure 5D). The control protein RalF did not co-purify with either H_6_IcmS or H_6_IcmW (Figure 5D). These data suggest that IcmS and IcmW each have effector binding activity.

LvgA is a factor that has been shown to contribute to the stability of IcmS and IcmW in L. pneumophila cells by interacting directly with IcmS [39,40]. SidG did not co-purify with H_6_LvgA when both proteins were produced in E. coli (Figure 5D). Additionally, translocation of Cya-SidG by an *lvgA* mutant was not as severely attenuated as that observed for Cya-SidG in the Δ*icmS* mutant (Figure S3). These data suggest that LvgA is not interacting with effectors directly to regulate translocation.

Deletion derivatives were analyzed to identify potential binding regions for the IcmSW complex on SidG. N-terminal deletion analysis revealed interactions between H_6_IcmW/IcmS and SidG*_(500–965)_* (Figure 5E). The SidG*_(600–965)_* protein did not interact with H_6_IcmW/IcmS, indicating that a binding region for IcmSW is located in the SidG*_(500–600)_* region. Interestingly, when C-terminal deletions were analyzed, an interaction between IcmSW and SidG*_(1–500)_* was observed (Figure 5E). This suggests the existence of a second IcmSW binding region located in the SidG*_(1–500)_* region. Further deletion analysis revealed that SidG*_(1–300)_* failed to bind IcmSW. These data suggest that a second binding region for IcmSW resides between residues 300 and 500. Collectively, these data suggest that the SidG*_(300–600)_* region contains at least two IcmSW binding sites (Figure 5F), the locations of which are consistent with translocation data indicating that regions outside of the C-terminal translocation signal sequence are the targets for IcmSW function.

### SidG Stability, Solubility, and Sub-Cellular Localization Is Not Altered by IcmSW

**Figure 6:**
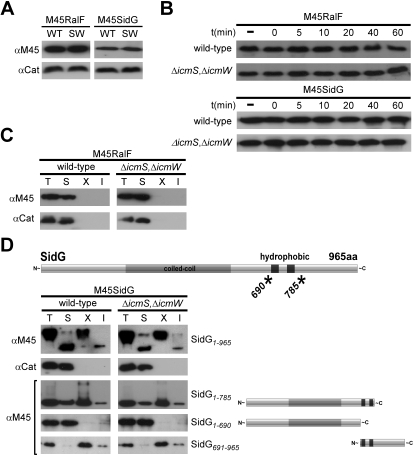
IcmSW Complex Does Not Influence the Stability, Solubility, or Sub-Cellular Localization of SidG (A) Immunoblots of whole-cell bacterial extracts expressing M45-SidG or M45-RalF proteins from wild-type (WT) or Δ*icmS*, Δ*icmW* (SW) probed with monoclonal antibody specific to the M45 epitope. Immunoblots for chloramphenicol acetyltransferase (Cat) are included as a load control. (B) Aliquots containing total protein from stationary phase wild-type or Δ*icmS*, Δ*icmW L. pneumophila* strains were precipitated (−). Cultures were harvested, resuspended in media containing kanamycin, and allowed to incubate at 37 °C for times indicated. Aliquots were mixed in sample buffer, resolved on SDS-PAGE, and detected with immunoblotting. (C) Wild-type or Δ*icmS*, Δ*icmW* mutant L. pneumophila expressing M45-RalF was cultured to stationary phase, harvested, lysed, and clarified (T). Ultracentrifugation separated a soluble (S) fraction from insoluble material. Inner membrane protein extraction with TritonX-100 (X) and subsequent ultracentrifugation separated this population from insoluble material (I). Protein contained in each fraction was detected with SDS-PAGE and immunoblotting. Immunoblots for (Cat) are included as a soluble protein control. (D) Wild-type or Δ*icmS*, Δ*icmW* mutant L. pneumophila expressing M45-SidG or indicated (*) SidG truncation proteins were cultured and subjected to sub-cellular fractionation as in (C). Protein contained in each fraction was detected with SDS-PAGE and immunoblotting. Immunoblots for (Cat) are included as a soluble protein control.

The binding of IcmSW to effectors suggests that this complex plays a role in maintaining full-length SidG in a translocation-competent state. One possible mechanism of IcmSW action would be to prevent deleterious interactions from occurring that prevent SidG from being properly engaged by the Dot/Icm system, which was investigated. In Figure 2C, the absence of the IcmSW complex was shown not have a dramatic effect on steady-state protein levels for the different Cya-Sid hybrids, suggesting that IcmSW is not important for preventing the degradation of effectors. Additionally, we found that IcmSW was not important for maintaining intracellular levels of M45-tagged SidG (Figure 6A), and the half-life of SidG was not affected in the L. pneumophila Δ*icmS*, Δ*icmW* double mutant (Figure 6B). These data indicate that the decrease in SidG translocation observed for mutants deficient in the IcmSW complex was not due to reduced cellular levels of SidG.

Fractionation of bacterial cells revealed that there was no difference in the sub-cellular localization or solubility of RalF or SidG in wild-type L. pneumophila compared to the Δ*icmS*, Δ*icmW* double mutant (Figure 6C and 6D). Some of the SidG protein from bacterial lysates was located in a pellet fraction and could be extracted from this fraction using TritonX-100. Deletion of the C-terminal 180 aa of SidG (SidG*_(1–785)_*) did not affect localization of the protein. When hydrophobic segments in SidG predicted to be membrane-spanning domains were deleted, the resulting SidG*_(1–690)_* protein was found exclusively in the soluble fraction, suggesting that the hydrophobic segments are capable of mediating membrane association (Figure 6D). The SidG*_(691–965)_* protein, which harbors the putative membrane-spanning regions, fractionated with TritonX-100-soluble membrane proteins when expressed in L. pneumophila, providing additional evidence that these hydrophobic regions mediate membrane interactions (Figure 6D). No differences for these SidG deletion derivatives were observed when sub-cellular localization of the proteins was compared in wild-type L. pneumopihla cells to Δ*icmS*, Δ*icmW* double mutant cells. Although these data suggest that SidG has the potential to associate with membranes, the IcmSW complex did not affect this localization.

To determine whether the absence of IcmSW results in SidG aggregation, gel filtration chromatography was used to compare the mobility of SidG produced in wild-type L. pneumophila to SidG produced in a Δ*icmS*, Δ*icmW* double mutant (see Protocol S1). SidG was not detected in the void volume when lysates from wild-type or Δ*icmS*, Δ*icmW* double mutant L. pneumophila were fractionated using a Superdex-200 column, suggesting that SidG protein aggregates were not present in the lysates (Figure S4). Interestingly, the mobility of SidG isolated from the Δ*icmS*, Δ*icmW* double mutant was slightly different from the mobility of SidG isolated from wild-type L. pneumophila. This could reflect a conformational difference in the SidG protein or this shift could be the direct result of IcmSW being associated with SidG. In addition to gel filtration, glycerol gradient centrifugation was used to examine whether SidG aggregates were present in the Δ*icmS*, Δ*icmW* double mutant (see Protocol S1). Consistent with the gel filtration results, glycerol gradients revealed no evidence of SidG aggregation in the Δ*icmS*, Δ*icmW* double mutant (Figure S4). Thus, we conclude that the reduction of SidG translocation in the Δ*icmS*, Δ*icmW* double mutant is not the result of rapid degradation of SidG, alterations in the solubility of SidG, or differences in the sub-cellular localization of SidG in the Δ*icmS*, Δ*icmW* double mutant. It is possible, however, that IcmSW binding alters the conformation of SidG, and that this could be important for engagement of SidG by the Dot/Icm system.

### IcmSW Binding Alters the Accessibility of Proteases to SidG

**Figure 7:**
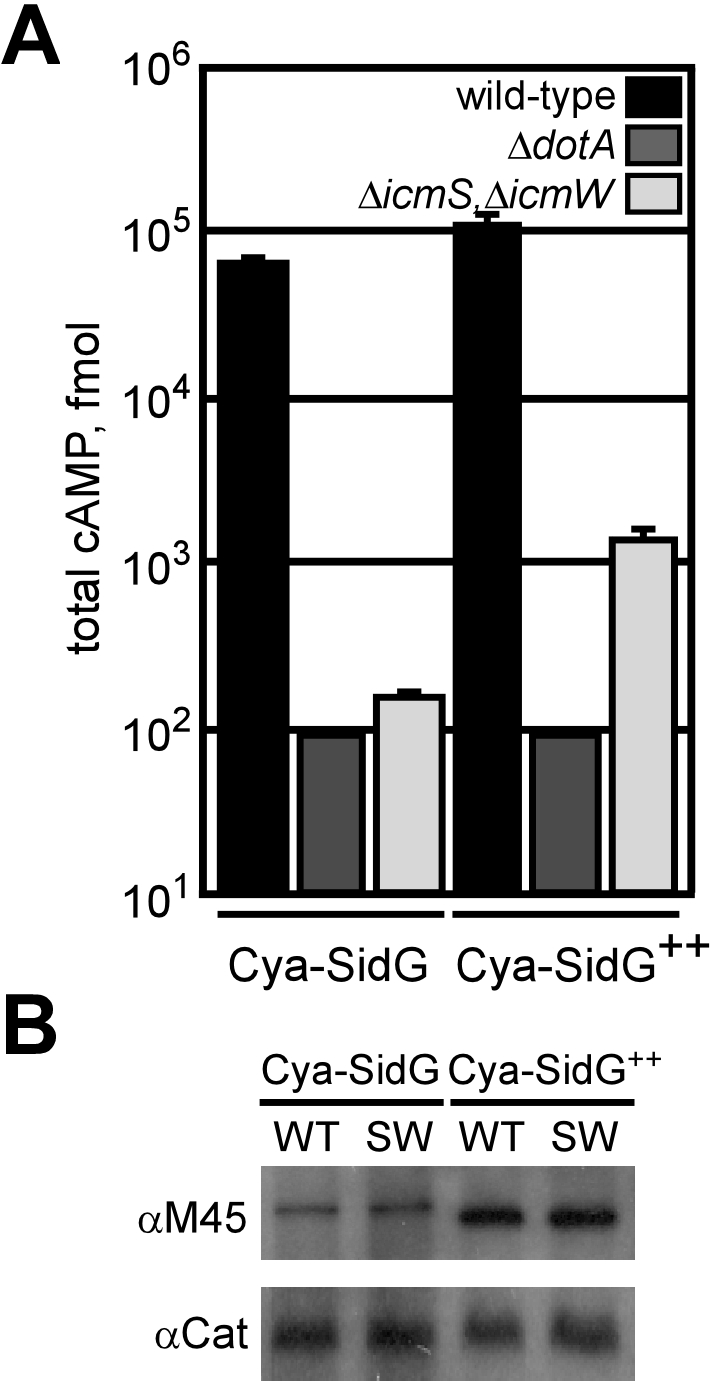
Overproduction of SidG Can Bypass the *icmSW* Requirement (A) CHO FcγRII cells were infected with wild-type (black), Δ*dotA* (dark grey), or Δ*icmS*, Δ*icmW* (light grey) L. pneumophila strains expressing the indicated Cya-SidG proteins for 1 h. Cultures that were grown in the presence of 1 mM IPTG prior to and during infection are indicated (Cya-SidG^++^). Total cAMP production induced by translocation of the hybrid was determined as in Figure 1. (B) Immunoblots of whole-cell bacterial extracts expressing Cya-SidG cultured in the absence or presence of 1 mM IPTG (Cya-SidG^++^). Proteins from wild-type (WT) or Δ*icmS*, Δ*icmW* (SW) were probed with monoclonal antibody specific to the M45 epitope. Immunoblots for chloramphenicol acetyltransferase (Cat) are included as a load control.

If the primary role of the IcmSW complex is to facilitate a C-terminal signal sequence being displayed by an effector protein, the percent decrease in translocation of an effector observed using a Δ*icmS*, Δ*icmW* double mutant would correlate with a drop in the ratio of effector in a conformational state where the C-terminal domain is accessible. Accordingly, increasing the total amount of SidG in the cell, which would increase the total number of SidG molecules in a conformational state where the C-terminal domain is accessible, should suppress the translocation defect in the Δ*icmS*, Δ*icmW* double mutant. By contrast, if the primary role of the IcmSW complex is to prevent effector proteins from aggregating, overproduction of the effector should augment the translocation defect. To determine whether overproduction of SidG had any effect on translocation by the Dot/Icm system, Cya-SidG production was increased using a heterologous IPTG-inducible promoter upstream of the gene encoding the reporter. After Cya-SidG production was upregulated by IPTG induction, translocation levels were measured and compared with previous translocation data. As shown in Figure 7, IPTG induction of the gene encoding Cya-SidG in the Δ*icmS*, Δ*icmW* double mutant resulted in a 10-fold increase in translocation compared to the control in which IPTG was not added. IPTG induction of the gene encoding Cya-SidG in the wild-type strain resulted in only a modest increase in translocation, indicating that the amount of SidG in a translocation-competent state is near saturation in the wild-type strain.

**Figure 8:**
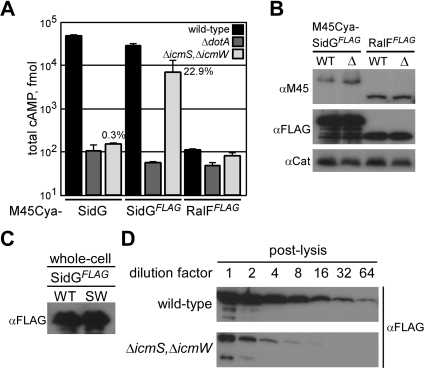
A C-Terminal FLAG Epitope Alters the Requirement for *icmSW* in SidG Translocation (A) CHO FcγRII cells were infected with wild-type (black), Δ*dotA* (dark grey), or Δ*icmS*, Δ*icmW* (light grey) strains expressing the indicated Cya hybrid proteins. Total cAMP production induced by translocation of the hybrid was determined as in (Figure 1). % values represent translocation efficiency as a measure of (Δ*icmS*, Δ*icmW*) / wild-type. (B) Immunoblots of whole-cell bacterial extracts expressing Cya-SidG*^FLAG^* or Cya-RalF*^FLAG^* proteins from wild-type (WT) or Δ*icmS*, Δ*icmW* (Δ) probed with monoclonal antibodies specific to the M45 or FLAG epitopes. Immunoblots for chloramphenicol acetyltransferase (Cat) are included as a load control. (C) Immunoblots of whole-cell bacterial extracts expressing SidG*^FLAG^* from wild-type (WT) or Δ*icmS*, Δ*icmW* (SW) probed with monoclonal antibody specific to the FLAG epitope. (D) SidG*^FLAG^* was expressed in wild-type or Δ*icmS*, Δ*icmW L. pneumophila* strains to stationary phase. Cultures were harvested, lysed in the absence of protease inhibitors, and clarified. Lysates were subjected to serial dilutions that were mixed with sample buffer and analyzed by SDS-PAGE and immunoblotting with monoclonal antisera specific for the FLAG epitope.

The hypothesis that IcmSW binding to effectors results in a conformational change that enhances recognition of the C-terminal translocation domain predicts that alterations in the C-terminal region of an effector could also reduce the requirement for IcmSW binding to an effector by enhancing presentation of the translocation domain. To address this possibility, an 8-aa FLAG epitope containing multiple charged residues was positioned at the C-terminus of RalF and SidG fusion proteins having M45-Cya at the N-terminus. The FLAG-tagged RalF and SidG proteins were expressed in either wild-type L. pneumophila or the Δ*icmS*, Δ*icmW* double mutant to assay translocation. In wild-type L. pneumophila the Cya-SidG*^FLAG^* protein was translocated to levels similar to those of the Cya-SidG protein, indicating that the C-terminal FLAG epitope does not affect SidG translocation (Figure 8A). In the Δ*icmS*, Δ*icmW* double mutant, translocation of the Cya-SidG*^FLAG^* protein was 100-fold higher than translocation of the control Cya-SidG protein, indicating that inclusion of an amino terminal FLAG tag greatly reduces the requirement for *icmSW*. The observation that translocation of the Cya-SidG*^FLAG^* protein was still reduced in the Δ*icmS*, Δ*icmW* double mutant compared to translocation by wild-type *L. pneumophila* indicates that the FLAG tag does not fully suppress the requirement for *icmSW*, which suggests that the tag does not disrupt the conformation of the SidG protein to the same extent as IcmSW binding. The Cya-RalF*^FLAG^* hybrid was not translocated to detectable levels, consistent with previous results demonstrating that the RalF C-terminal translocation domain is non-functional when masked by an epitope tag (Figure 7B) [32]. Both RalF and SidG hybrids were detected at similar levels from wild-type L. pneumophila and the Δ*icmS*, Δ*icmW* double mutant using antiserum specific for either the N-terminal M45 epitope or the C-terminal FLAG epitope (Figure 8B), suggesting that protein stability was not altered significantly by addition of the C-terminal FLAG tag.

**Figure 9:**
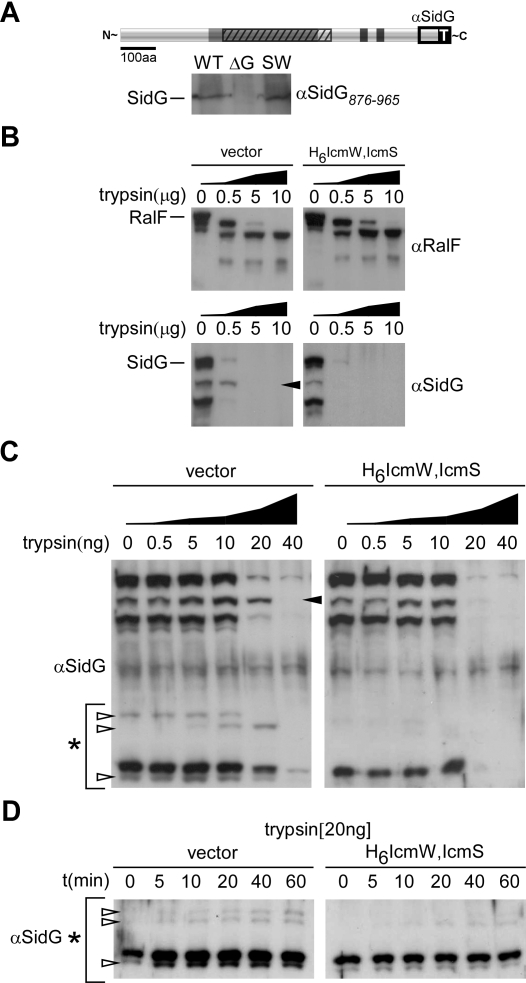
IcmSW Binding to SidG Promotes Conformational Changes in the Effector Protein (A) Schematic representation of the SidG polypeptide, with the IcmSW binding domain shown (hatch) as well as the indicated C-terminal domain (876–965) used for generation of polyclonal antisera. Full-length SidG was detected at equivalent levels from wild-type (WT) or Δ*icmS*, Δ*icmW* mutant L. pneumophila (SW) and was not detected in the *sidG* deletion strain (ΔG). (B) Recombinant RalF or SidG were co-expressed in the absence (vector) or presence of the IcmSW complex (H_6_IcmW,IcmS), and subjected to lysis and clarification. Aliquots of soluble whole-cell lysates were subjected to the indicated concentrations of trypsin for 1 h. Samples were subjected to SDS-PAGE and immunoblotting, where proteolytic fragments of RalF (RalF−) or SidG (SidG−) were detected with specific antisera raised against the indicated polypeptides. The black arrow indicates predominant cleavage product. (C) Lysates containing SidG were processed as in (B), except that trypsin was diluted 1,000-fold. Proteolytic cleavage fragments (white arrows) emerge in the absence of IcmSW in the molecular size range of 50–60 kDa as indicated by the asterisk. (D) Proteolytic time course experiment performed at indicated concentration (20 ng) on SidG-containing lysates. Immunoblots show differential cleavage products (arrows) in the 50–60 kDa molecular size range (*).

Expression of SidG*^FLAG^* with the Cya domain removed resulted in equivalent amounts of steady-state protein levels detected in wild-type L. pneumophila and the Δ*icmS*, Δ*icmW* double mutant when total protein was precipitated from whole cells (Figure 8C). When bacteria were lysed in the absence of protease inhibitors, however, the SidG*^FLAG^* protein produced by the Δ*icmS*, Δ*icmW* double mutant showed signs of degradation by an endogenous protease (Figure 8D). This result suggested that IcmSW binding to SidG*^FLAG^* promotes a conformational change in the substrate that affects protease sensitivity following bacterial disruption. A polyclonal antibody specific for the C-terminal 90 aa of the SidG polypeptide was used to examine whether the binding of IcmSW to SidG results in a conformational change that can be detected by protease accessibility. This antibody detected endogenous SidG produced by wild-type L. pneumophila and showed that endogenous SidG levels were similar in the Δ*icmS*, Δ*icmW* double mutant (Figure 9A), providing further evidence that the IcmSW complex is not required for SidG stability.

To determine whether IcmSW binding alters protease accessibility, RalF and SidG were produced in E. coli in either the absence or presence of the H_6_IcmW/IcmS complex. Cleavage products from whole cell lysates partially digested with trypsin were detected by immunoblot analysis using antibodies specific for RalF or SidG (Figure 9B). Cleavage products observed for RalF were indistinguishable in the presence and absence of the H_6_IcmW/IcmS complex. These data indicate that IcmSW does not affect RalF conformation and are consistent with data showing that IcmSW does not bind to RalF. Differences in the appearance of a predominant SidG cleavage product were observed when reactions containing IcmSW were compared to reactions without IcmSW, which is consistent with the hypothesis that IcmSW binding alters the conformation of SidG (Figure 9B). Similar assays were performed on SidG lysates where the trypsin was diluted 1,000-fold. A difference in the appearance of a major cleavage product was again observed when reactions containing IcmSW were compared to reactions without IcmSW (Figure 9C). Importantly, cleavage products in a size range between 50 kDa and 60 kDa were detected in reactions without the IcmSW complex, and were less prominent in reactions containing the IcmSW complex. Because the SidG antibody recognizes the C-terminal 90-aa region of the protein, the locations of the cleavage sites must be located between residues 400 and 500 of SidG. These data are consistent with IcmSW interactions with SidG protecting protease-sensitive sites in the region required for IcmSW binding. To more carefully examine the emergence of these cleavage products, a time course experiment was performed with a constant protease concentration (Figure 9D). Again, SidG cleavage products in the 50- to 60-kDa range preferentially emerge in lysates that do not contain IcmSW. Collectively, these results indicate that IcmSW alters the conformation of SidG, and suggest that binding of IcmSW to the central core of SidG protects protease sensitive sites.

## Discussion

Over 50 different L. pneumophila Dot/Icm effectors have been identified, and the total number of effectors is expected to exceed 150 [12–26]. Secondary structure predictions indicate that L. pneumophila effectors are diverse (Figures S1 and 6D). There are effectors with predicted membrane-spanning helices, regions of coiled-coil domains, and homologies found primarily among eukaryotic proteins. How such a large number of effectors with such diverse structural properties can be efficiently recognized by a single secretion system is a question of fundamental importance. Analysis of the RalF protein has shown that substituting individual amino acids at positions near the C-terminus can dramatically reduce Dot/Icm-mediated translocation [32], which is consistent with studies of other type IV secretion systems where individual amino acid residues critical for type IV translocation have been identified near the C-terminus [29]. The C-terminal 20-aa residues that comprise the translocation signal for the RalF protein were disordered in the X-ray crystal structure [35]. This has led to speculation that a disordered C-terminal domain could be important for effector protein engagement with the type IV secretion machinery. In addition to a C-terminal translocation signal, the IcmSW complex had been implicated as being important for recognition of effector proteins by the Dot/Icm system [21]. Here, we provide data that link the function of the IcmSW complex with the hypothesis that a disordered C-terminal secretion signal is important for effector protein translocation.

Our data show that IcmSW can bind to effectors, that the binding region is located outside of the C-terminal translocation domain, and that Dot/Icm-mediated recognition of the minimal C-terminal translocation domain does not require the IcmSW complex (Figures 3, 4, and 5). These data are inconsistent with the IcmSW complex functioning as an adapter that facilitates recognition of a C-terminal translocation domain by binding to both the effector and the Dot/Icm translocation apparatus. If the primary function of IcmSW was to serve as an adapter, then removing the binding site for IcmSW from an effector should result in a translocation defect in wild-type L. pneumophila that is comparable with the translocation defect observed for the full-length effector produced in the Δ*icmS*, Δ*icmW* double mutant. Our data show the opposite. We found that removing regions from the effector SidG that contained binding sites for the IcmSW complex eliminated a requirement for the IcmS and IcmW proteins for efficient translocation (Figure 4). Taken together, these data identify internal regions of an effector protein that have a negative effect on recognition of the C-terminal translocation signal and show that IcmSW binding to these regions neutralizes this negative effect.

There are two likely explanations for why IcmSW binding to an effector can neutralize intrinsic signals that interfere with translocation. The first is that IcmSW binding prevents inappropriate interactions between the effector and other proteins. Alternatively, IcmSW binding could prevent interactions from occurring within the effector that masks recognition by the Dot/Icm system. We were unable to show that effectors are more prone to aggregation or degradation in the absence of the IcmSW complex, which would be effects that might result from inappropriate homo- or heterotypic protein–protein interactions (Figures 6 and S4). The observation that overproduction of the effector protein SidG in an Δ*icmS*, Δ*icmW* double mutant suppressed the translocation defect also suggests that the IcmSW complex is not preventing inappropriate homo- or heterotypic protein–protein interactions, as overexpression would most likely promote these interactions and augment the translocation defect (Figure 7).

Importantly, we were able to show that introduction of a small epitope tag to the SidG C-terminus greatly affected the *icmSW* dependency for translocation, and enhanced susceptibility of SidG*^FLAG^* to degradation following lysis of Δ*icmS*, Δ*icmW* double mutant *L. pneumophila*, suggesting that IcmSW binding alters the conformation of SidG (Figure 8). This evidence was further supported by limited proteolysis experiments that demonstrated conformational changes in SidG induced by IcmSW binding. Thus, our data fit better with a model that predicts IcmSW binding to effectors facilitates the display of the C-terminal translocation domain (Figure S5).

The model we propose also suggests that the IcmSW complex binds to effectors before the proteins have folded completely. Whether IcmSW binding to effectors is co-translational or occurs after synthesis has been completed can not be determined from our data; however, the observation that purified IcmSW does not bind in vitro to effectors expressed separately suggests that IcmSW binds poorly to effectors in their native conformation. Importantly, previous data has shown that IcmS binds very well to members of the SidE family of effectors after transfer from a SDS-PAGE gel to a nitrocellulose membrane, suggesting that IcmS can bind to an immobilized effector in a denatured state [25,36]. These data also imply that IcmS can bind to an effector independent of IcmW. Consistent with these previous data, we show here that IcmS and IcmW can bind to the effector protein SidG independent of each other and that binding can be detected only when the proteins are synthesized in the same bacterial cell (Figure 5). Although effectors may have independent binding sites for IcmS and IcmW, the observation that IcmW is highly unstable when it is not associated with IcmS implies that these proteins function as a complex [21].

It is significant that IcmS and IcmW are relatively small proteins predicted to have an acidic pI, which are properties shared by chaperone proteins that function in the translocation of effector proteins by a type III secretion mechanism [37]. The model we propose for the function of the IcmSW complex has parallels to what is known about the function of chaperones used for effectors translocated through the type III secretion apparatus. Chaperones used by the type III secretion system maintain effectors in a partially unfolded state, which facilitates recognition of an N-terminal secretion signal in type III effectors [42–46]. Recognition of the effector by the type III secretion system stimulates disassembly of the chaperone–effector complex by an ATPase that is essential for secretion system function [44,47]. Our data suggest that IcmSW binding to effectors facilitates display of a C-terminal translocation signal. There are at least two proteins in the Dot/Icm system with predicted nucleotide-binding sites that might engage effectors and initiate translocation through the Dot/Icm system [39,48]. In particular, the DotB protein is a critical component of the Dot/Icm system that is thought to hydrolyze ATP to energize transport of effectors through the apparatus [48]. It will be interesting to test in the future whether DotB can recognize C-terminal translocation signals directly and has an activity that disassembles the IcmSW–effector protein complex.

The crystal structure of RalF and molecular analysis of this protein have provided clues as to why not all Dot/Icm effectors show the same level of dependency for IcmSW for translocation [35]. RalF does not bind to IcmSW, the IcmSW complex does not contribute to RalF translocation, and the RalF protein has a disordered C-terminal translocation signal. According to the RalF data, effectors having a C-terminal translocation domain that is disordered in the absence of IcmSW binding should be engaged by the Dot/Icm system efficiently by mutants lacking the IcmSW complex. This would explain why appending a minimal C-terminal translocation sequence to Cya results in Dot/Icm-dependent translocation by a process that is independent of IcmSW. According to our model of IcmSW function, removal of sequences that are N-terminal to the translocation signal of an *icmSW*-dependent effector prevents complete folding of the C-terminal domain containing this signal, mimicking what occurs when IcmSW binds to the full-length effector.

Although the model we have put forward suggests that IcmSW binding to effectors facilitates presentation of a C-terminal translocation domain, it is possible that IcmSW binding serves other functions that are more subtle and difficult to reveal using the assays available to measure effector protein translocation and our limited knowledge of the type IV secretion pathway. For instance, if type IV effectors are translocated in a partially unfolded state, IcmSW binding could potentially be important for translocation of effectors that are difficult for the type IV machinery to unfold once they have achieved their most thermodynamically stable conformation. Additionally, with over 150 predicted effectors, it remains unclear how translocation of effectors is regulated and whether spatial and temporal regulation of effector translocation is important for L. pneumophila modulation of host cell functions [26,39,49,50]. Furthermore, IcmSW could compete with inhibitory proteins that sequester substrates under unfavorable conditions for translocation. Whether IcmSW binding to effectors plays any role in regulating the timing of effector translocation remains an interesting possibility. As more information on the molecular mechanisms important for type IV secretion become available, it should be possible to test how IcmSW might affect recognition of effectors by other type IV secretion components. In conclusion, these studies on IcmSW function have provided new insight into how effector protein C-terminal translocation domains are presented to the type IV machinery and indicate that future studies on this interesting chaperone complex should reveal new information on biochemical activities important for effector protein translocation through the type IV apparatus.

## Materials and Methods

### Bacterial strains and plasmids.

Bacterial strains, plasmids, and primers used to generate constructs in this study are described in Table S1. The *sidG* deletion strain was generated as described, using the primers 69,70,71,103 [21]. L. pneumophila strains were cultivated on charcoal yeast-extract agar (CYEA) or ACES yeast-extract broth (AYE) (pH 6.9) supplemented with chloramphenicol (6.25 μg/ml), or kanamycin (20 μg/ml) where appropriate. E. coli strains were cultivated in Luria-Bertani (LB) broth supplemented with chloramphenicol (25 μg/ml), or ampicillin (100 μg/ml) where appropriate.

### Cya translocation assay.

Translocation assays were performed as described [21]. Briefly, monolayers containing 1 × 10^5^ CHO FcγRII cells were infected with 3 × 10^6^ opsonized L. pneumophila (MOI = 30) expressing Cya hybrid proteins. After 1 h of incubation at 37 °C, 5% CO_2_, monolayers were washed with PBS and lysed. Total cAMP was extracted and quantified using cAMP Biotrak Enzymeimmunoassay System (GE Healthcare). Where appropriate, cytochalasin D (10 μM) (Sigma-Aldrich) was added to the tissue culture medium 30 min prior to and throughout the infection.

### Immunoblotting.

Stationary phase L. pneumophila were harvested, corrected for load (OD_600_), and precipitated in 10% trichloroacetic acid. Precipitated protein was washed in acetone, resuspended in sample buffer (62.5 mM Tris-HCl [pH 6.8], 20% SDS, 20% glycerol, 8% β-mercaptoethanol, 3 M urea), boiled, and loaded on 10% or 15% SDS-PAGE. Gels were transferred to PVDF, and blocked and probed with antibody specific for either the M45 epitope, DotA, RalF, chloramphenicol acetyltransferase (Cat) (Sigma-Aldrich), or FLAG (Sigma-Aldrich). Detection was carried out as described [21]. The SidG polyclonal antibody was generated against purified recombinant H_6_SidG*_876–965_* by Cocalico Biologicals.

### Phagocytosis assay.

Monolayers of 1 × 10^5^ CHO FcγRII cells were cultured in medium containing L. pneumophila–specific opsonizing antibody for 1 h prior to infection. Where appropriate, cytochalasin D was added to (10 μM) 30 min prior to infection. L. pneumophila strains were added to the monolayer (MOI = 30), centrifuged 5 min at 1,000 rpm, heated to 37 °C in a water bath for 5 min, and subjected to incubation at 37 °C, 5% CO_2_ for 1 h. Gentamycin (Invitrogen) was added (10 μM) 15 min after infection where appropriate. Media was aspirated from the monolayers and they were washed 3x with PBS. Tissue culture cells were osmotically lysed with ice-cold H_2_0 for 10 min. Lysates were resuspended and serially diluted, and aliquots were plated on CYEA and incubated at 37 °C to determine colony-forming units (CFU).

### Co-purification of the IcmSW complex with effectors.

E. coli BL21-DE3 (Novagen) was transformed with an expression vector (pEC66) that harbors a di-cistronic message encoding H_6_ *icmW* and *icmS*, as well as a second expression vector that encodes an N-terminal M45 epitope–tagged hybrid to the L. pneumophila gene of interest (RalF or SidG). Cultivation and co-purification of complexes were performed as described [21]. To examine the capture of SidG by H_6_IcmW/IcmS, 0.5-l cultures of E. coli BL21-DE3 expressing H_6_IcmW and IcmS were cultivated either together or separately from an expression vector producing M45-SidG. Cultures were harvested, resuspended in 22 ml of lysis buffer (50 mM Tris-HCl [pH 7.5], 150 mM NaCl, 1 mM DTT, 1 mM PMSF), and subjected to lysis by French pressure cell (single pass at 18,000 × *psi*). Cultures were clarified by centrifugation at 15,000*g* for 15 min, and 20 ml of the supernatant was loaded onto a Ni-NTA column (1 ml bed volume) (Qiagen) equilibrated with lysis buffer. When produced separately, clarified lysates from E. coli BL21-DE3 expressing M45-SidG were loaded on Ni-NTA columns that were pre-charged with lysates expressing H_6_IcmW/IcmS and washed with 20 ml lysis buffer. Full-length and truncation derivatives of M45-SidG that were co-expressed with H_6_IcmW/IcmS were lysed, clarified, and loaded onto Ni-NTA columns under identical conditions. Columns were washed with lysis buffer containing 10 mM imidazole, 5 mM MgATP (20 ml), 25 mM imidazole (10 ml), 50 mM imidazole (10 ml), and eluted with 500 mM imidazole (4 × 1 ml) in lysis buffer (fraction 2 shown). Each fraction is represented at equivalent volumes with the exception of the eluate fractions, which are enriched 5x. Detection of the IcmSW component was verified by mixing the eluate 1:1 with sample buffer, resolving on 15% SDS-PAGE and Coomassie blue staining. Detection of M45-tagged protein was visualized through immunoblotting the fractions with monoclonal antisera specific to the M45 epitope. Co-purification of SidG or RalF with either H_6_IcmS (pEC27), H_6_IcmW (pEC13), or H_6_LvgA (pEC329) was performed similarly.

### Pulse-chase analysis.

L. pneumophila strains expressing N-terminal M45 epitope–tagged effectors were cultured in AYE broth at 37 °C to stationary phase, corrected for bacterial load by absorbance at 600 nm (10 ml of OD_600_ = 4), and harvested by centrifugation. Samples were aspirated and pellets were resuspended in 10 ml of pre-warmed AYE broth (37 °C) supplemented with kanamycin (50 μg/ml). Cultures were incubated in a 37 °C water bath, where aliquots of the suspension were collected at incremental time points, precipitated in 10% TCA, washed in acetone, resuspended in sample buffer, and analyzed by SDS-PAGE and immunoblotting.

### Sub-cellular fractionation.

L. pneumophila strains expressing M45 epitope–tagged effectors (Ralf or SidG) were cultured in AYE broth at 37 °C to stationary phase, corrected for bacterial load by absorbance at 600 nm (10 ml of OD_600_ = 4), and harvested by centrifugation. Bacterial pellets were resuspended in 0.5 ml ice-cold 200 mM Tris-HCl (pH 8.0) and 0.5 ml ice-cold 50 mM Tris-HCl (pH 8.0), 1 M sucrose). Then, 10 μl of 0.5 M EDTA (pH 8.0) and 10 μl of 10 mg/ml lysozyme (Invitrogen) were added to the suspension, which was diluted to a final volume of 2 ml with ice-cold H_2_O. After 30 min on ice, MgSO_4_ was added to a final concentration of 20 mM. Spheroplasts were harvested by centrifugation at 5,000*g* for 10 min, and resuspended in 5 ml 50 mM Tris-HCl (pH 8.0). Samples were subjected to lysis by sonication (3 × 30-sec bursts at 25%–35% intensity) and were centrifuged at 5,000*g* for 10 min to produce a clarified lysate. The clarified lysate was removed and subjected to ultracentrifugation at 100,000*g* for 1 h (4 °C). The supernatant fraction was separated from the pellet, and the pellet fraction was resuspended in 1 ml Tris-HCl (pH 8.0). 10% TritonX-100 (in H_2_O) was next added to the resuspended pellet fraction to achieve a final concentration of 1% (vol/vol) and incubated on ice for 30 min. Suspensions were subjected to ultracentrifugation at 100,000*g* for 1 h (4 °C). The supernatant fraction was separated from the pellet fraction, which was resuspended in 1.1 ml Tris-HCl (pH 8.0). Each of the four fractions were mixed 1:1 with sample buffer, and subjected to 10% SDS-PAGE. Fractions were equivalently loaded on gels based on the volume of buffer that the fraction received. Samples were transferred to PDVF membrane and probed with monoclonal antisera specific to the N-terminal M45 epitope tag.

### Limited proteolysis.

E. coli BL21-DE3 (Novagen) was transformed with an expression vector alone (pET15b) or pEC66 that harbors a di-cistronic message encoding H_6_ *icmW* and *icmS*, as well as a second expression vector that encodes an N-terminal M45 epitope–tagged hybrid to the L. pneumophila gene of interest (RalF or SidG). Stationary phase cultures were diluted 1:50 in 0.5 l and incubated on a shaker (275 rpm) for 2 h at 37 °C. IPTG was added to a final concentration of 1 mM and cultures were cultivated an additional 3 h at 37 °C. Cultures were harvested, resuspended in 10 ml of lysis buffer (50 mM Tris-HCl [pH 7.5], 150 mM NaCl, 1 mM DTT), and subjected to lysis by French pressure cell (single pass at 18,000 × *psi*). Cultures were clarified by centrifugation at 15,000*g* for 15 min and supernatants were adjusted for total protein concentration by absorbance at 280 nm and dilution with lysis buffer. Next, 100-μl aliquots were added to 40 μl lysis buffer containing 0, 0.5, 5, 10, 20, or 40 μg trypsin (Sigma-Aldrich) on ice. Experments performed at the μg scale were incubated on ice for 1 h, whereas experiments performed at the ng scale were incubated at 37 °C for 1 h. After 1 h, PMSF was added to each sample to a final concentration of 1 mM. Time course experiments were performed similarly, where each aliquot was subjected to 20 ng trypsin and reactions were stopped with 1 mM PMSF. Each 140-μl reaction was next mixed with 100 μl sample buffer, boiled, and subjected to SDS-PAGE and immunoblotting with polyclonal antisera generated against either RalF or SidG*_786–965_*.

## Supporting Information

Figure S1 Dot/Icm Secretion Substrates Display Diverse Molecular Character (A) The Sid proteins utilized for this study are highlighted (bold-type). Each substrate is drawn to scale as a measure of primary amino acid sequence length, with regions containing coiled-coil homology stretches shown in blue, and hydrophobic segments shown in red. Several Sid proteins contain paralogous copies on the *L. pneumophila* genome (grey). A 42-kDa RalF, which contains a Sec7 homology domain (yellow), is included for size perspective.
(B) CHO FcγRII cells were infected with wild-type (black) or Δ*dotA* (grey) L. pneumophila strains expressing the indicated Cya hybrid proteins. After 1 h of infection, tissue culture cells were lysed and cAMP was extracted from the sample. Total cAMP production induced by translocation of the hybrid was quantified using an enzyme-immunoassay system, indicated as fmol. Results represent average values ± SD of experiments performed in triplicate.
(97 KB PDF)

Figure S2 Mapping the *icmSW* Requirement for SidG Translocation (A) Schematic representation of indicated SidG truncation hybrids. Each truncation was fused in frame to the C-terminal end of Cya. Relative amino acid positions of SidG hybrids are indicated on the right. *icmS/W*-independent translocation was observed for each hybrid highlighted in green, while *icmS/W*-dependent translocation was observed for each hybrid highlighted in red.
(B) CHO FcγRII cells were infected with wild-type (black), Δ*dotA* (dark grey), or Δ*icmS*, Δ*icmW* (light grey) L. pneumophila strains expressing the indicated Cya-SidG hybrid proteins for 1 h. Total cAMP production induced by translocation of the hybrid was determined as in Figure S1.
(100 KB PDF)

Figure S3 Contribution of *lvgA* to the Translocation of RalF and SidG (A) CHO FcγRII cells were left uninfected (hatch) or infected with wild-type (black), *lvgA*::TnNH3503 (dark grey), Δ*icmS* (light grey), or Δ*icmS*, Δ*icmW* (white) L. pneumophila strains expressing the indicated Cya hybrid proteins for 1 h. Total cAMP production induced by translocation of the hybrid was determined as in Figure S1.
(40 KB PDF)

Figure S4 SidG Does Not Form Aggregates When Expressed in Δ*icmS*, Δ*icmW L. pneumophila*
(A) M45-SidG was expressed in wild-type or Δ*icmS*, Δ*icmW L. pneumophila* strains and cultured to stationary phase. Cultures were harvested, lysed, clarified, and subjected to gel filtration chromatography on a Superdex-200 column. Total protein was loaded at equivalent levels (L). Peak elution of SidG expressed in the Δ*icmS*, Δ*icmW* strain eluted earlier than of that expressed in wild-type (dotted lines); however, no high molecular weight SidG aggregates were detected.
(B) M45-SidG was expressed in wild-type or Δ*icmS*, Δ*icmW L. pneumophila* strains and cultured to stationary phase. Cultures were harvested, lysed, clarified, and subjected to a 15%–45% glycerol gradient. Fractions collected from the gradient were subjected to SDS-PAGE and immunoblotting with antisera specific for the M45 epitope to detect SidG migration. DotA and RalF were detected with specific monoclonal and polyclonal antisera, respectively.
(46 KB PDF)

Figure S5 Model Depicting the Contribution of IcmSW to the Recognition of Effector Proteins by the Dot/Icm Transporter in L. pneumophila
IcmS (red) and IcmW (blue) form a complex in the L. pneumophila cytoplasm. Each factor harbors a binding activity on effectors, where the complex might capture nascent chains emerging from ribosomes prior to effector protein folding. IcmSW binding promotes a conformational change in the effector that may facilitate presentation of the C-terminal translocation domain (green) to the Dot/Icm machinery.
(88 KB PDF)

Protocol S1 Supplemental Materials and Methods (28 KB DOC)

Table S1 Bacterial Strains, Plasmids, and Primers Employed in This Study (157 KB DOC)

## Abbreviations

aa: amino acid(s)
cAMP: cyclic adenosine-monophosphate
Cya: adenylate cyclase
H_6_IcmW: hexa-histidine-tagged IcmW
*sid,*: substrate of *icm-dot* transporter
T4SS: type IV secretion system
