# Supplementary material for: The Legionella pneumophila IcmSW Complex Interacts with Multiple Dot/Icm Effectors to Facilitate Type IV Translocation
Source: PLoS Pathog. 2007 Dec 7;3(12):e188. doi: 10.1371/journal.ppat.0030188 (PMC2134951; doi:10.1371/journal.ppat.0030188)
Supplement: Supplementary file 1 [file ppat.0030188.sg001.pdf]

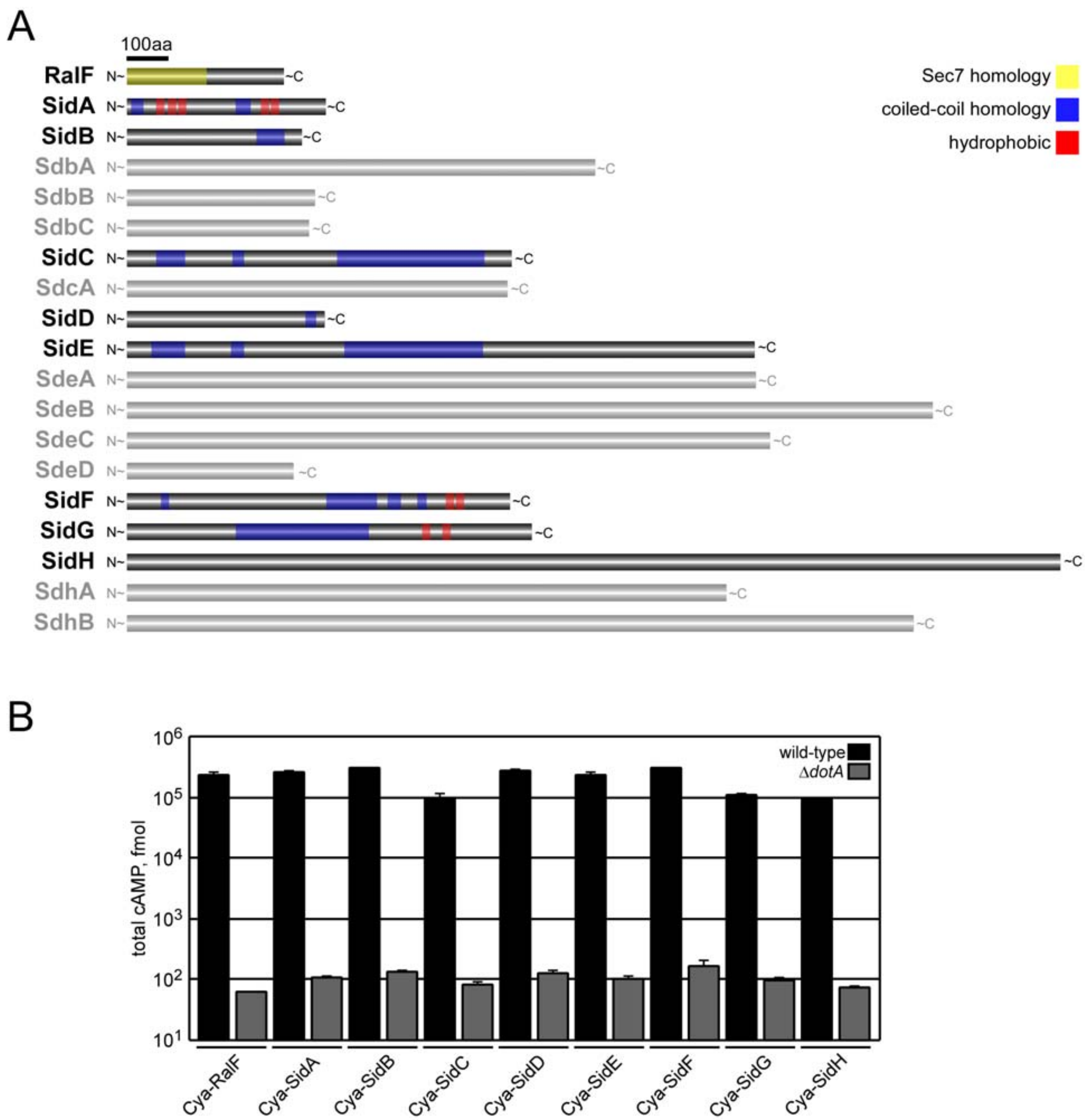
A
100aa
RalF
SidA
SidB
SdbA
SdbB
SdbC
SidC
SdcA
SidD
SidE
SdeA
SdeB
SdeC
SdeD
SidF
SidG
SidH
SdhA
SdhB
Sec7 homology
coiled-coil homology
hydrophobic
B
total cAMP, fmol
wild-type
ΔdotA
Cya-RalF
Cya-SidA
Cya-SidB
Cya-SidC
Cya-SidD
Cya-SidE
Cya-SidF
Cya-SidG
Cya-SidH
