# Supplementary material for: The Legionella pneumophila IcmSW Complex Interacts with Multiple Dot/Icm Effectors to Facilitate Type IV Translocation
Source: PLoS Pathog. 2007 Dec 7;3(12):e188. doi: 10.1371/journal.ppat.0030188 (PMC2134951; doi:10.1371/journal.ppat.0030188)
Supplement: Supplementary file 2 [file ppat.0030188.sg002.pdf]

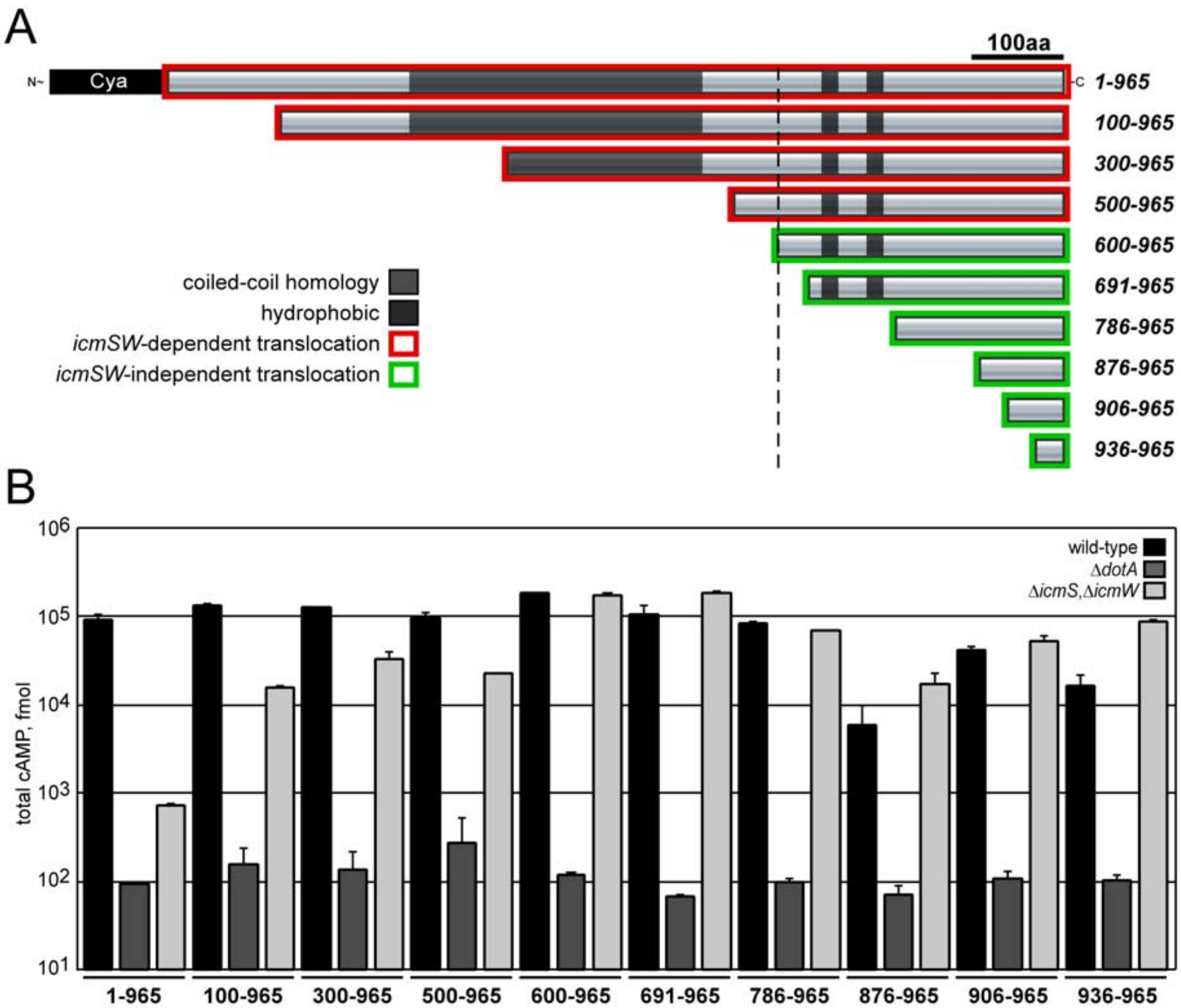
A
100aa
N~
Cya
C
1-965
100-965
300-965
500-965
600-965
691-965
786-965
876-965
906-965
936-965
coiled-coil homology
hydrophobic
icmSW-dependent translocation
icmSW-independent translocation
B
total cAMP, fmol
wild-type
ΔdotA
ΔicmS,ΔicmW
10^6
10^5
10^4
10^3
10^2
10^1
1-965
100-965
300-965
500-965
600-965
691-965
786-965
876-965
906-965
936-965
