# Supplementary material for: The Legionella pneumophila IcmSW Complex Interacts with Multiple Dot/Icm Effectors to Facilitate Type IV Translocation
Source: PLoS Pathog. 2007 Dec 7;3(12):e188. doi: 10.1371/journal.ppat.0030188 (PMC2134951; doi:10.1371/journal.ppat.0030188)
Supplement: Supplementary file 3 [file ppat.0030188.sg003.pdf]

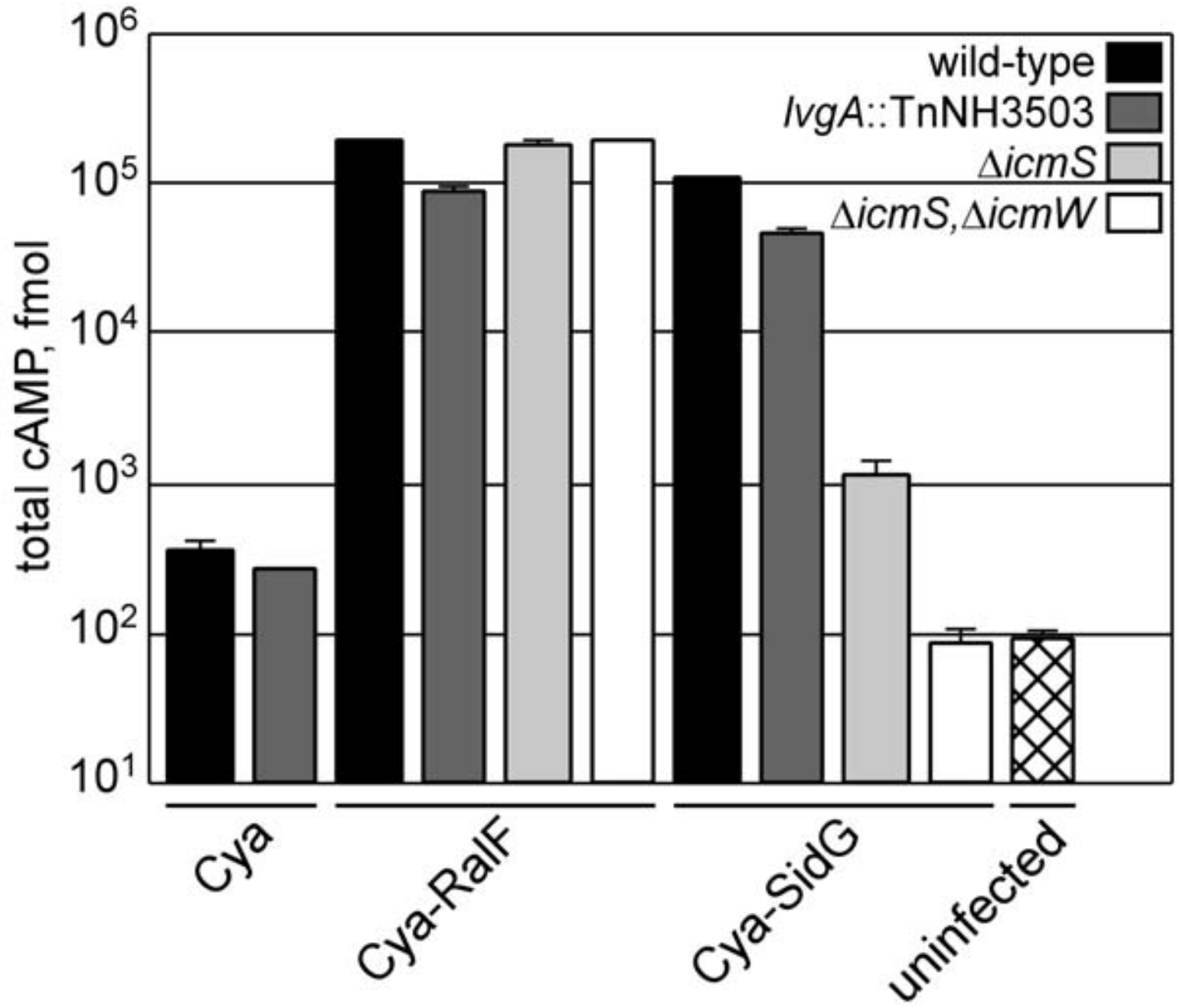

10^6
10^5
10^4
10^3
10^2
10^1
total cAMP, fmol
wild-type
lvgA::TnNH3503
ΔicmS
ΔicmS,ΔicmW
Cya
Cya-RalF
Cya-SidG
uninfected
