# Supplementary material for: The Legionella pneumophila IcmSW Complex Interacts with Multiple Dot/Icm Effectors to Facilitate Type IV Translocation
Source: PLoS Pathog. 2007 Dec 7;3(12):e188. doi: 10.1371/journal.ppat.0030188 (PMC2134951; doi:10.1371/journal.ppat.0030188)
Supplement: Supplementary file 4 [file ppat.0030188.sg004.pdf]

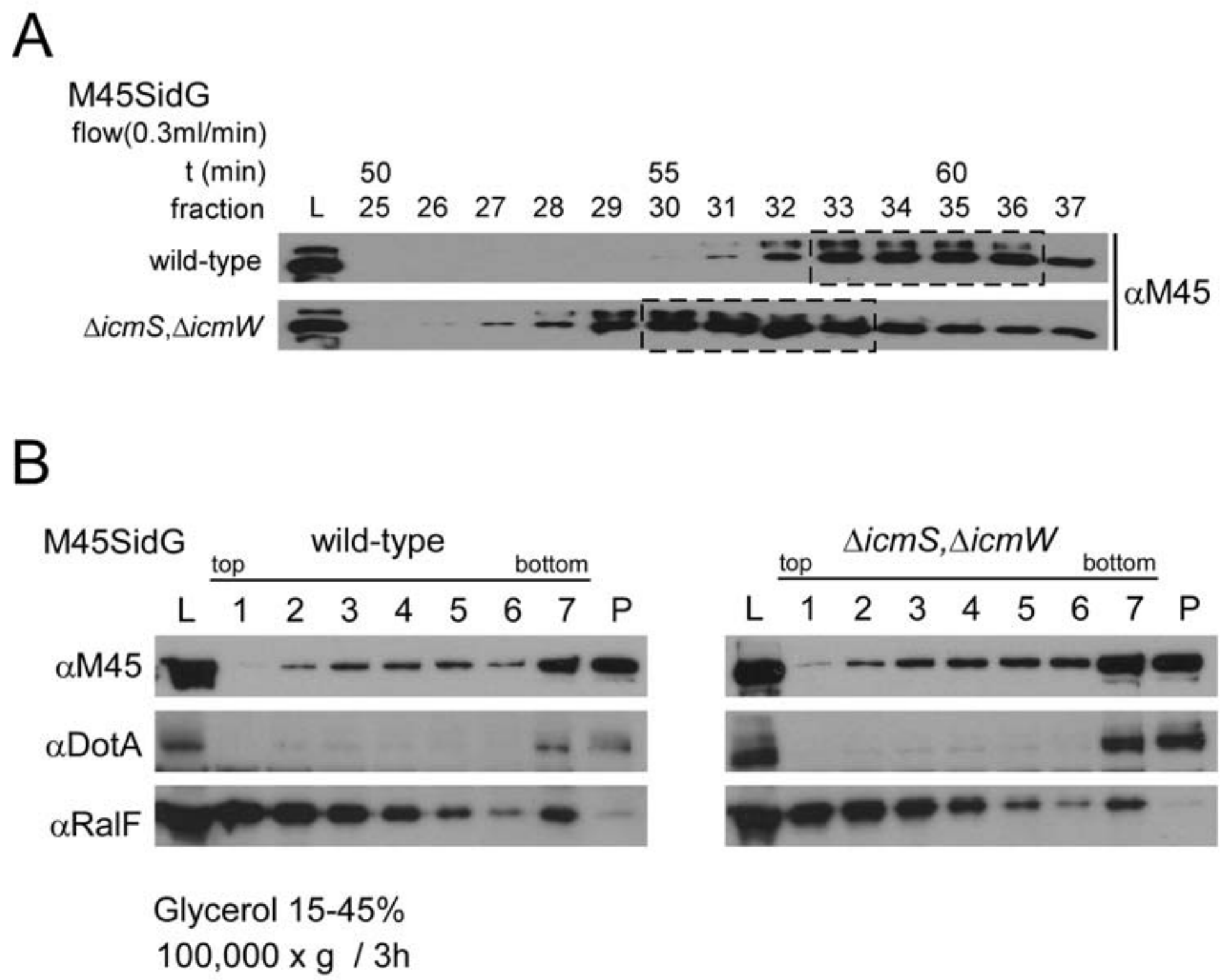
A
M45SidG
flow(0.3ml/min)
t (min) 50 55 60
fraction L 25 26 27 28 29 30 31 32 33 34 35 36 37
wild-type
ΔicmS,ΔicmW
αM45
B
M45SidG
wild-type
top bottom
L 1 2 3 4 5 6 7 P
ΔicmS,ΔicmW
top bottom
L 1 2 3 4 5 6 7 P
αM45
αDotA
αRalF
Glycerol 15-45%
100,000 x g / 3h
