# Supplementary material for: The Legionella pneumophila IcmSW Complex Interacts with Multiple Dot/Icm Effectors to Facilitate Type IV Translocation
Source: PLoS Pathog. 2007 Dec 7;3(12):e188. doi: 10.1371/journal.ppat.0030188 (PMC2134951; doi:10.1371/journal.ppat.0030188)
Supplement: Supplementary file 5 [file ppat.0030188.sg005.pdf]

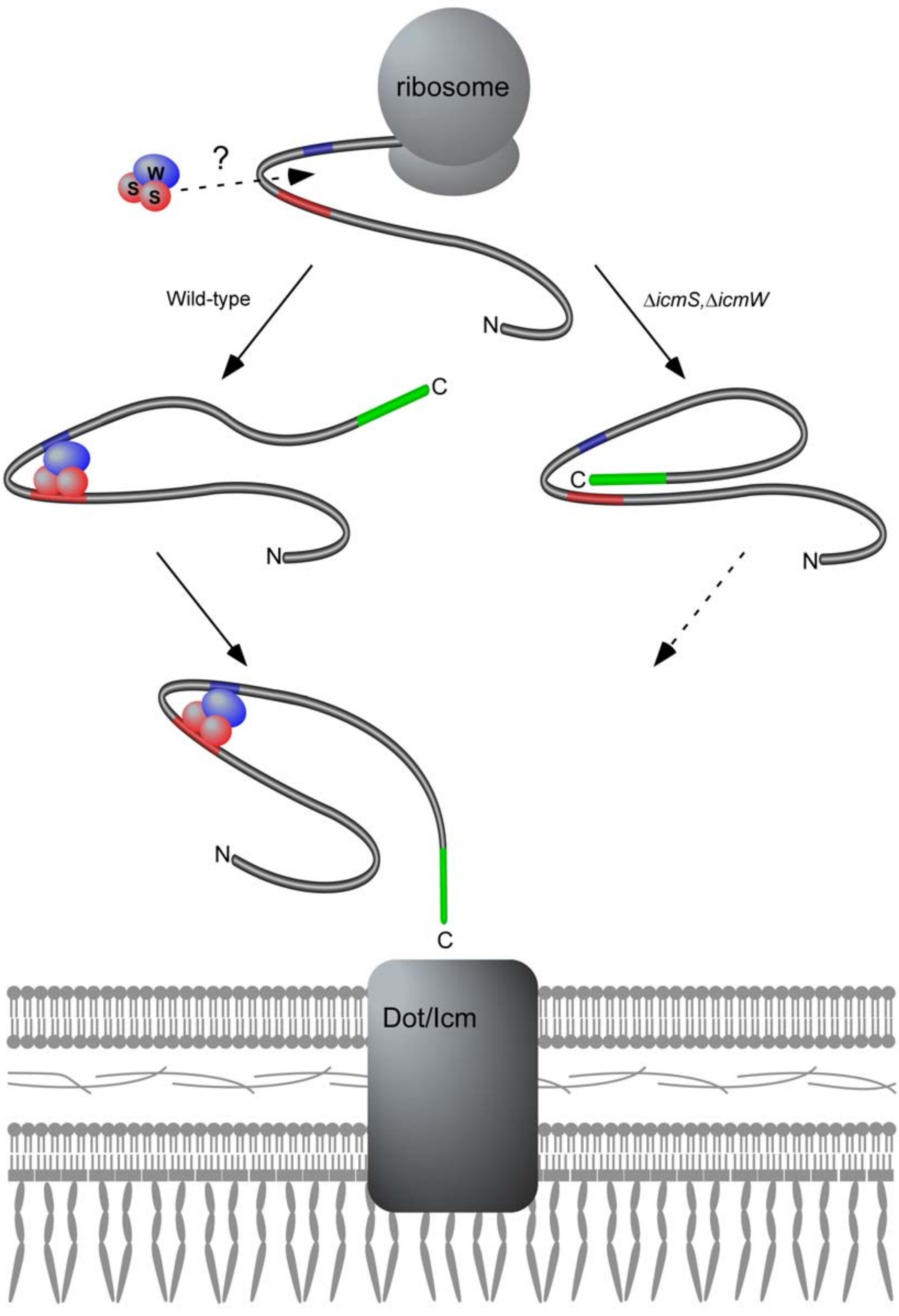
ribosome
W
S
S
?
Wild-type
ΔicmS,ΔicmW
N
C
N
C
N
N
C
Dot/Icm
