# Supplementary material for: The Legionella pneumophila IcmSW Complex Interacts with Multiple Dot/Icm Effectors to Facilitate Type IV Translocation
Source: PLoS Pathog. 2007 Dec 7;3(12):e188. doi: 10.1371/journal.ppat.0030188 (PMC2134951; doi:10.1371/journal.ppat.0030188)
Supplement: Supplementary file 6 [file ppat.0030188.sd001.doc]

**Supplemental materials and methods**

**Gel filtration chromatography.** *L. pneumophila* strains expressing M45 epitope-tagged SidG were cultured in 100ml AYE broth at 37ºC to stationary phase and harvested by centrifugation. Bacterial pellets were resuspended in 10ml lysis buffer (30mM Tris-HCl, pH8.0, 200mM NaCl, 1mM DTT, 1 x complete protease inhibitor [Roche]) and subjected to lysis by French pressure cell (single pass at 18,000*psi*). Lysates were clarified by centrifugation at 15,000 x *g* for 15min and corrected for total protein load by absorbance at 280nm, adjusting volumes with lysis buffer. 500μl of equilibrated clarified lysates from individual strains were injected onto a Superdex-200 FPLC column (GE Healthcare) equilibrated with lysis buffer. Fractions [300μl] were collected over the entire course of the experiment, where aliquots were mixed 1:1 with sample buffer and subjected to 10% SDS-PAGE. Samples were transferred to PDVF membrane and probed with monoclonal antisera specific to the N-terminal M45 epitope-tagged SidG.

**Glycerol gradient centrifugation.** *L. pneumophila* strains expressing M45 epitope-tagged SidG were cultured in 100ml AYE broth at 37ºC to stationary phase and harvested by centrifugation. Bacterial pellets were resuspended in 10ml lysis buffer (50mM Tris-HCl, pH7.5, 150mM NaCl, 1mM DTT, 1 x complete protease inhibitor [Roche]) and subjected to lysis by French pressure cell (single pass at 18,000 x *psi*). Lysates were clarified by centrifugation at 15,000 x *g* for 15min and corrected for total protein load by absorbance at 280nm, adjusting volumes with lysis buffer. 70μl of equilibrated clarified lysates from individual strains were loaded onto a 700μl 15-45% glycerol gradient (produced from step-wise 100μl aliquots of 5% decreasing intervals of glycerol mixed with 50mM Tris-HCl, pH7.5, 150mM NaCl, 1mM DTT). Samples were subjected to ultracentrifugation on a fixed angle rotor for 3hr at 100,000 x *g*. Fractions were collected in 110μl aliquots from top to bottom of the column. The pellet fraction was resuspended in 110μl of lysis buffer. Each fraction was suspended 1:1 in sample buffer, boiled and subjected to 10% SDS-PAGE. Samples were transferred to PDVF membrane and probed with monoclonal antisera specific to the N-terminal M45 epitope-tag of SidG, or DotA, or with polyclonal antisera specific for RalF.

**Accession numbers for the *L.pneumophila* proteins employed in this study.** Proteins examined in this study may be accessed at NCBI / Genbank with the following accession numbers: SidA (AAR90347), SidB (AAR90348), SdbA (AAR90349), SdbB (AAR90350), SdbC (AAR90351), SidC (AAR90352), SdcA (AAR90353), SidD (AAR90354), SidE (AAR90355), SdeA (AAR90356), SdeB (AAR90357), SdeC (AAR90358), SdeD (AAR90359), SidF (AAR90360), SidG (AAR90361), SidH (AAR90362), SdhA (AAR90363), SdhB (AAR90364), and RalF (AAL23711).
