# Supplementary material for: The Legionella pneumophila IcmSW Complex Interacts with Multiple Dot/Icm Effectors to Facilitate Type IV Translocation
Source: PLoS Pathog. 2007 Dec 7;3(12):e188. doi: 10.1371/journal.ppat.0030188 (PMC2134951; doi:10.1371/journal.ppat.0030188)
Supplement: Supplementary file 7 [file ppat.0030188.st001.doc]

| **Table S1. Bacterial strains, plasmids, and primers employed in this study** | | | | |
| --- | --- | --- | --- | --- |
| Bacterial Strains | | Genotype |  | Reference |
| *L. pneumophila* | |  |  |  |
| CR39 | | serogroup 1, strain LP01 *rpsL* |  | [1] |
| CR58 | | LP01 Δ*dotA* *rpsL* |  | [1] |
| CR157 | | LP01 Δ*icmW* *rpsL* |  | [2] |
| CR393 | | LP01 Δ*icmS* *rpsL* |  | [3] |
| CR503 | | LP01 Δ*icmS,*Δ*icmW* *rpsL* |  | [3] |
| EC1 | | LP01 Δ*sidG*, *rpsL* |  | This study |
| 52B12 | | LP01 *lvgA*::TnNH3503 *rpsL* |  | [4] |
| *E. coli* | |  |  |  |
| DH5α | | *supE44* Δ*lacU169 (*Φ80*lacZ*Δ*M15) hsdR17 recA1 endA1 gyrA96 thi-1 relA1* |  | [5] |
| BL21-DE3 | | *E.coli* B F^-^ *dcm ompT* *hsdS*(r**_B_**^-^ m**_B_**^-^) gal λ(DE3) |  | [6] |
| Plasmids | | Properties | Primers | Reference |
| pCya | | pMMB207NT.*cya* |  | [7] |
| pCya-RalF | | pMMB207NT.*cya.ralF* |  | [7] |
| pEC11 | | pMMB207NT.*cya.sidH* |  | [8] |
| pEC13 | | pET15b-*H_6_icmW* | 13,14 | This study |
| pEC19 | | pMMB207NT.*cya.sidA* | 26,27 | This study |
| pEC21 | | pMMB207NT.*cya.sidB* | 28,29 | This study |
| pEC23 | | pMMB207NT.*cya.sidC* | 30,31 | This study |
| pEC25 | | pMMB207NT.*cya.sidD* | 32,33 | This study |
| pEC27 | | pET15b-*H_6_icmS* | 10,12 | This study |
| pEC28 | | pMMB207NT.*cya.sidE* | 34,35 | This study |
| pEC31 | | pMMB207NT.*cya.sidF* | 36,37 | This study |
| pEC35 | | pMMB207NT.*cya.sidG* |  | [8] |
| pEC47 | | pMMB207NT.*sidG* |  | [8] |
| pEC66 | | pET15b-*H_6_icmW*-RBS-*icmS* |  | [8] |
| pEC101 | | pMMB207NT.*cya.sidG(876-965)* | 6,94 | This study |
| pEC103 | | pMMB207NT.*cya.sidG(906-965)* | 6,95 | This study |
| pEC112 | | pMMB207NT.*cya.sidG(786-965)* | 6,93 | This study |
| pEC114 | | pMMB207NT.*cya.sidG(936-965)* | 6,52 | This study |
| pEC129 | | pMMB207NT.*cya.sidG(100-965)* | 6,104 | This study |
| pEC131 | | pMMB207NT.*cya.sidG(300-965)* | 6,105 | This study |
| pEC133 | | pMMB207NT.*cya.sidG(500-965)* | 6,106 | This study |
| pEC135 | | pMMB207NT.*cya.sidG(691-965)* | 6,76 | This study |
| pEC183 | | pMMB207NT.*cya.sidG(946-965)* | 6,148 | This study |
| pEC184 | | pMMB207NT.*cya.sidG(956-965)* | 149,150 | This study |
| pEC185 | | pMMB207NT.*cya.sidG(961-965)* | 151,152 | This study |
| pEC188 | | pMMB207NT.*cya.sidG(1-935)* | 21,147 | This study |
| pEC201 | | pMMB207NT.*sidG(500-965)* | 6,106 | This study |
| pEC202 | | pMMB207NT.*sidG(691-965)* | 6,76 | This study |
| pEC203 | | pMMB207NT.*sidG(300-965)* | 6,105 | This study |
| pEC204 | | pMMB207NT.*sidG(786-965)* | 6,93 | This study |
| pEC205 | | pMMB207NT.*sidG(876-965)* | 6,94 | This study |
| pEC207 | | pMMB207NT.*sidG(1-500)* | 21,173 | This study |
| pEC208 | | pMMB207NT.*sidG(1-600)* | 21,172 | This study |
| pEC209 | | pMMB207NT.*sidG(1-690)* | 21,75 | This study |
| pEC211 | | pMMB207NT.*sidG(600-965)* | 6,171 | This study |
| pEC214 | | pMMB207NT.*cya.sidG(1-785)* | 21,174 | This study |
| pEC232 | | pMMB207NT.*sidG(1-785)* | 21,174 | This study |
| pEC247 | | pMMB207NT.*cya.sidG(1-920)* | 21,196 | This study |
| pEC248 | | pMMB207NT.*cya.sidG(1-930)* | 21,195 | This study |
| pEC249 | | pMMB207NT.*cya.sidG(1-940)* | 21,194 | This study |
| pEC277 | | pMMB207NT.*cya.sidG(1-965)^FLAG^* | 21,246 | This study |
| pEC278 | | pMMB207NT.*cya.ralF(1-374)^FLAG^* | 205,247 | This study |
| pEC279 | | pMMB207NT.*sidG(1-965)^FLAG^* | 21,246 | This study |
| pEC280 | | pMMB207NT.*ralF(1-374)^FLAG^* | 205,247 | This study |
| pEC299 | | pET15b-*sidG(876-965)* | 47,263 | This study |
| pEC329 | | pET15b-*H_6_lvgA* | 290,291 | This study |
| pEC343 | | pMMB207NT.*sidG(1-300)* | 21,316 | This study |
| pM45RalF | | pMMB207NT.*ralF* |  | [7] |
| Primer | Sequence | | | Site |
| 6 | CCTT**CTGCAG**TTATATGCGCATCGTACTTTCAG | | | *Pst*I |
| 10 | AA**GGATTC**CTAATCATACATTAACTCATCCA | | | *Bam*HI |
| 12 | AA**CATATG**GAGCGAGATATTAGCAAG | | | *Nde*I |
| 13 | AA**GGATCC**TTATTCATCCCCTTCGAGTGC | | | *Bam*HI |
| 14 | AA**CATATG**CCTGATTTAAGCCATGAAG | | | *Nde*I |
| 21 | CG**AGATCT**TTATGTCCCGTTCGAAAGATGAAG | | | *Bgl*II |
| 26 | CG**GGATCC**TTATGTCCAAAAAATATCGAACTCC | | | *Bam*HI |
| 27 | ATT**CTGCAG**TTAACGAGTTGGTTTGGATATGG | | | *Pst*I |
| 28 | CG**GGATCC**TTATGGCTAAAATTTATAATGCAC | | | *Bam*HI |
| 29 | ATT**CTGCAG**CTAATTTATTTCTGGTATACTTT | | | *Pst*I |
| 30 | CG**GGATCC**TTATGGTGATAAACATGGTTGACG | | | *Bam*HI |
| 31 | ATT**CTGCAG**CTATTTCTTTATAATTCCCGTG | | | *Pst*I |
| 32 | CG**GGATCC**TTATGCGTTCGATTATTACACAAATC | | | *Bam*HI |
| 33 | TTG**AAGCTT**TTAAATAGTAAGACTCGAGTTAG | | | *Pst*I |
| 34 | CG**GGATCC**TTATGCCTAAGTACGTTGAAGGGATAG | | | *Bam*HI |
| 35 | ATT**CTGCAG**TTACAATCTTAAGAGAGTTCCTTG | | | *Pst*I |
| 36 | CG**GGATCC**TTATGCCACGAATCACTGAAAATATAG | | | *Bam*HI |
| 37 | ATG**AAGCTT**TTAGAAGTTTACTGGCGTGGGG | | | *Hind*III |
| 47 | AA**AGATCT**TTATATGCGCATCGTACTTTCAG | | | *Bgl*II |
| 52 | CG**GGATCC**TTGAACCATCGATGAGCTCTAC | | | *Bam*HI |
| 69 | CAGTTCCAGTTTCTAAAACTTCATCTTTCGAAC | | | oligo |
| 70 | AGTTTTAGAAACTGGAACTGAAAGTACGATG | | | oligo |
| 71 | AA**TCTAGA**GAGCTTTATATAATTTGCTATTTC | | | *Xba*I |
| 75 | AA**CTGCAG**TTAGTATATTGACATGGCATATCCT | | | *Pst*I |
| 76 | AA**AGATCT**TTTACATGGATCCTGAAGCCTCA | | | *Bgl*II |
| 93 | CG**GGATCC**TTCTTATAGCAAATTTATTTAAACAAAA | | | *Bam*HI |
| 94 | CG**GGATCC**TTGTCCTAAAATTTGTATCAAAAAGT | | | *Bam*HI |
| 95 | CG**GGATCC**TTTTCGCACCCAAGACTAGGG | | | *Bam*HI |
| 103 | AA**GAGCTC**CCAAATTCACTTGTGCCTTGC | | | *Sac*I |
| 104 | CG**AGATCT**TTGTAGAGTTAAAAACGCAAGTTAC | | | *Bgl*II |
| 105 | CG**AGATCT**TTACACAAGTTAAAAATAAAGTCGAA | | | *Bgl*II |
| 106 | CG**AGATCT**TTGTTATATCAGAATCCTCTGTAAA | | | *Bgl*II |
| 147 | AA**CTGCAG**TTATTGTCTGGGACTTGTAGTTAAT | | | *Pst*I |
| 148 | CG**GGATCC**TTGAAGAAAACATCGATTCAGAAC | | | *Bam*HI |
| 149 | **GATCCTT**GAAACTGGAACTGAAAGTACGATGCGCATATAA**CTGCA** | | | oligo |
| 150 | **G**TTATATGCGCATCGTACTTTCAGTTCCAGTTTC**AAG** | | | oligo |
| 151 | **GATCCTT**AGTACGATGCGCATATAA**CTGCA** | | | oligo |
| 152 | **G**TTATATGCGCATCGTACT**AAG** | | | oligo |
| 171 | CG**AGATCT**TTGGTCCAGAAAAAAAAGAATTCAA | | | *Bgl*II |
| 172 | AA**CTGCAG**TTAAGCTACAAGCTCATGCATTTTT | | | *Pst*I |
| 173 | AA**CTGCAG**TTATTGGCATGGAGAGTCAATTTTG | | | *Pst*I |
| 174 | AA**CTGCAG**TTATTTAAATGGCTTGGCTATCGTT | | | *Pst*I |
| 194 | AA**CTGCAG**TTAGCTCATCGATGGTTCTTGTC | | | *Pst*I |
| 195 | AA**CTGCAG**TTAAGTTAATTCCGTTTTATCAGTAG | | | *Pst*I |
| 196 | AA**CTGCAG**TTATTCTTCCACCAATTCATCAATTT | | | *Pst*I |
| 205 | AA**GGATCCTT**CATCCAGAAATTGAAAAAGCCC | | | *Bam*HI |
| 246 | CG**CTGCAG**TTACTTGTCATCGTCGTCCTTGTAGTCTATGCGCATCGTACTTTCAGT | | | *Pst*I |
| 247 | CG**CTGCAG**TTACTTGTCATCGTCGTCCTTGTAGTCAAATTTTAATTGTCTACCTTTTTCT | | | *Pst*I |
| 263 | AA**CATATG**GTCCTAAAATTTGTATCAAAAAGT | | | *Nde*I |
| 290 | AA**CATATG**GCAGACGGCGATATCGAAAT | | | *Nde*I |
| 291 | AA**GGATCC**TTATTTTCGTGCAGTAGTTGCA | | | *Bam*HI |
| 316 | AA**CTGCAG**TTATGAGTCTACCTCTTTTTTAGC | | | *Pst*I |

**References**

1. Berger KH, Isberg RR (1993) Two distinct defects in intracellular growth complemented by a single genetic locus in *Legionella pneumophila*. Mol Microbiol 7: 7-19.

2. Zuckman DM, Hung JB, Roy CR (1999) Pore-forming activity is not sufficient for *Legionella pneumophila* phagosome trafficking and intracellular growth. Mol Microbiol 32: 990-1001.

3. Coers J, Kagan JC, Matthews M, Nagai H, Zuckman DM, et al. (2000) Identification of Icm protein complexes that play distinct roles in the biogenesis of an organelle permissive for *Legionella pneumophila* intracellular growth. Mol Microbiol 38: 719-736.

4. Murata T, Delprato A, Ingmundson A, Toomre DK, Lambright DG, et al. (2006) The *Legionella pneumophila* effector protein DrrA is a Rab1 guanine nucleotide-exchange factor. Nat Cell Biol 8: 971-977.

5. Hanahan D (1983) Studies on transformation of *Escherichia coli* with plasmids. J Mol Biol 166: 557-580.

6. Studier FW, Moffatt BA (1986) Use of bacteriophage T7 RNA polymerase to direct selective high-level expression of cloned genes. J Mol Biol 189: 113-130.

7. Nagai H, Cambronne ED, Kagan JC, Amor JC, Kahn RA, et al. (2005) A C-terminal translocation signal required for Dot/Icm-dependent delivery of the *Legionella* RalF protein to host cells. Proc Natl Acad Sci U S A 102: 826-831.

8. Ninio S, Zuckman-Cholon DM, Cambronne ED, Roy CR (2005) The *Legionella* IcmS-IcmW protein complex is important for Dot/Icm-mediated protein translocation. Mol Microbiol 55: 912-926.
